# Supplementary material for: Cytoskeletal Control of Antigen-Dependent T Cell Activation
Source: Cell Rep. 2019 Mar 19;26(12):3369–3379.e5. doi: 10.1016/j.celrep.2019.02.074 (PMC6426652; doi:10.1016/j.celrep.2019.02.074)
Supplement: Document S1. Figures S1–S4 [file mmc1.pdf]

Cell Reports, Volume *26*

Supplemental Information

Cytoskeletal Control of Antigen-Dependent T Cell Activation

Huw Colin-York, Yousef Javanmardi, Mark Skamrahl, Sudha Kumari, Veronica T. Chang, Satya Khuon, Aaron Taylor, Teng-Leong Chew, Eric Betzig, Emad Moeendarbary, Vincenzo Cerundolo, Christian Eggeling, and Marco Fritzsche

Supplementary Figures

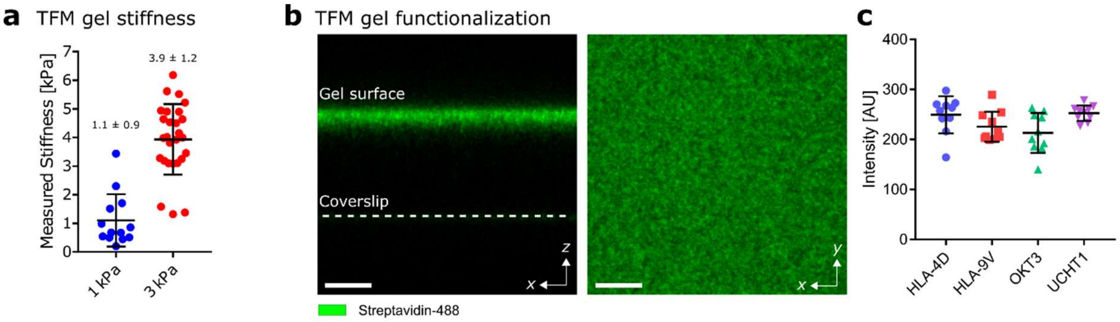

Figure S1 – **PAA Gel characterisation,** Related to Figure 1 - a) Stiffness of 1 kPa and 3 kPa PAA gel as measured by AFM indentation. b) Antigen coating on the PAA gel labelled by streptavidin 488. Scale bar is 10 µm. c) Quantification of antigen coating on the top surface of the gel. Scale bar is 5 µm.

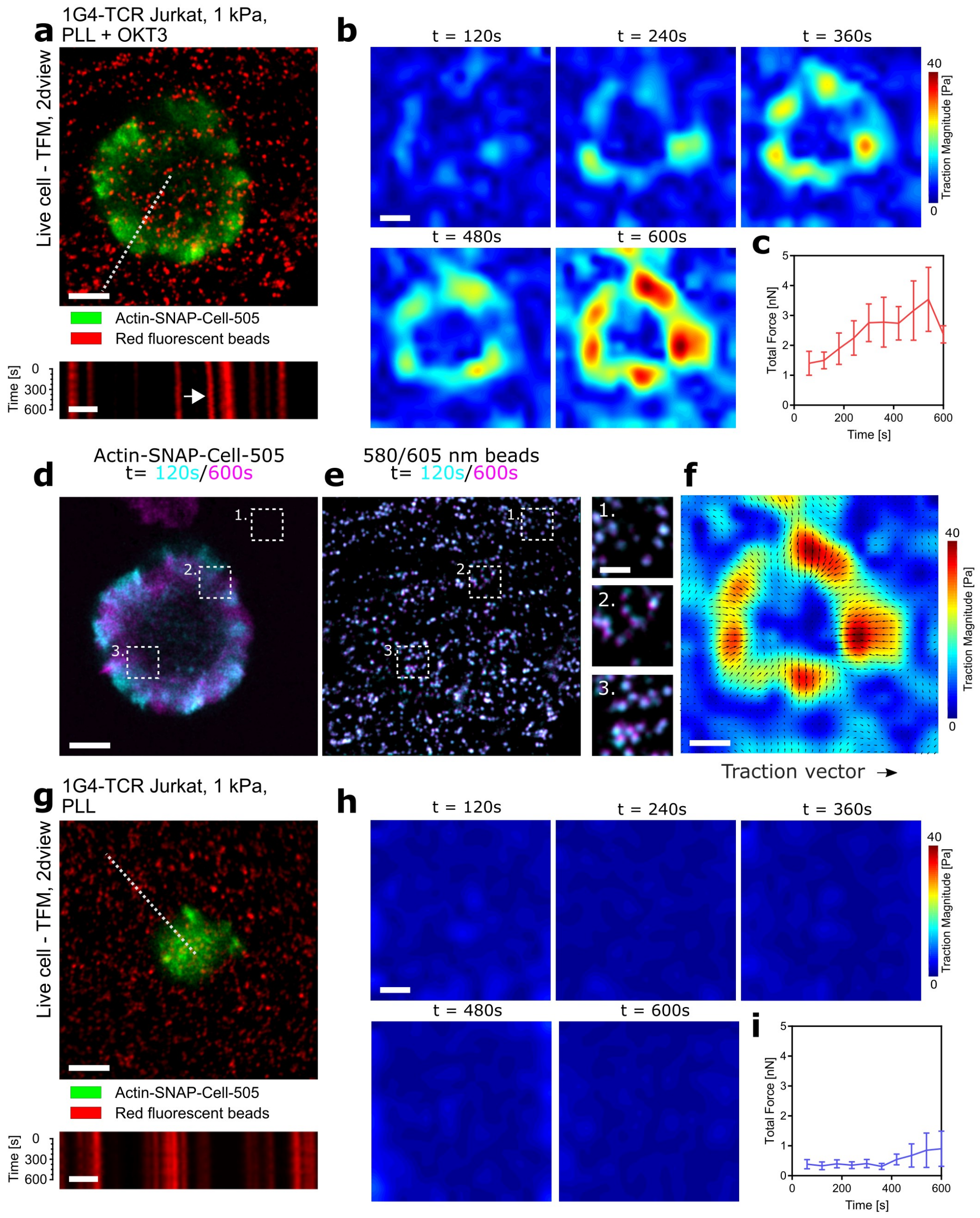

Figure S2 - **Force generation in the presence of OKT3 and PLL**, Related to Figure 1 - a) Upper - 1G4 TCR Jurkat T-cell interacting with 1 kPa PAA gel functionalised with PLL and OKT3. Scale bar is 5 µm. Lower – kymograph showing the gradual bead displacement during synapse maturation. Scale bar is 2 µm. b) Cumulative traction magnitude during the interaction, showing the gradual increase in force as the synapse matures in the presence of PLL and OKT3. Scale bar is 5 µm. c) Plot displays the integrated force over the whole contact area and again illustrates the gradual increase in the forces over the course of 600 seconds. d) Distribution of fluorescent actin during the early (cyan) and late (magenta) stage of synapse formation. Scale bar is 5 µm. e) Fluorescent bead distribution under the cell during the early (cyan) and late (magenta) stage of synapse formation. Inset dotted regions are shown on the right, and the lateral displacement of the beads is evident, showing the generation of force by the cell. Scale bar is 2 µm. f) Traction magnitude overlay with a traction vector field for the 1G4 TCR Jurkat T-cell interacting with 1 kPa PAA gel functionalised with PLL and OKT3. g) Upper - 1G4 TCR Jurkat T-

cell interacting with 1 kPa PAA gel functionalised with PLL only. Scale bar is 5 µm. Lower - kymograph showing minimal bead displacement during cell contact. Scale bar is 2 µm. h) Cumulative traction magnitude during the interaction, showing no force generation of the duration of the contact. Scale bar is 5 µm. i) Plot displays the integrated force over the whole contact area and again illustrates the lack of force generation over the course of 600 seconds.

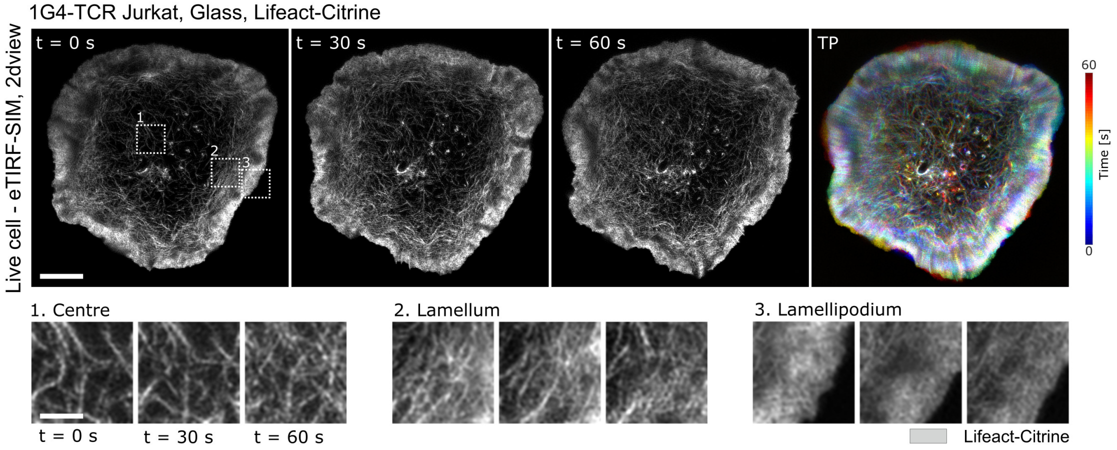

Figure S3 - **Actin dynamics imaged by eTIRF-SIM**, Related to Figure 2 – eTIRF-SIM timelapse imaging of 1G4-TCR Jurkat T-cells expressing Lifeact-**Citrine** interacting with antigen coated coverslips together with temporal projection (TP). Scale bar is 5 µm. Lower shows actin architecture in the central, lamellum and lamellipodial region of the cell. Scale bar is 1 µm.

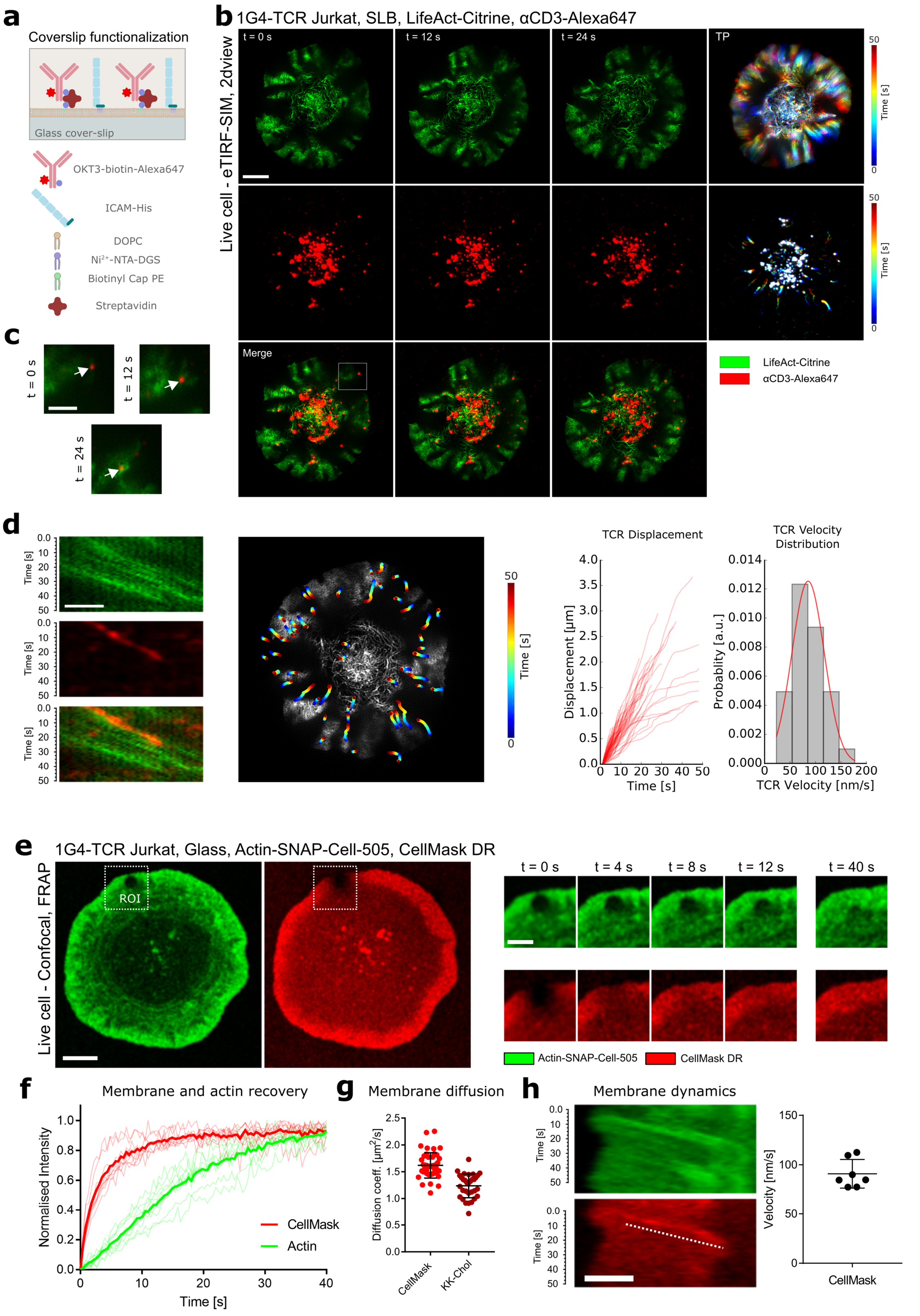

Figure S4 – **TCR cluster and membrane dynamics during T-cell activation,** Related to Figure 3 - a) Schematic outlining the functionalisation of SLB coated coverslips allowing stimulation via anti-CD3 antibodies. b) eTIRF-SIM fluorescent imaging time-lapse of 1G4-TCR Jurkat T-cell expressing Lifeact-citrine (green) interacting with

an SLB coated coverslips coated with anti-CD3-Alexa647 (red) and temporal projection (TP). Scale bar is 5 μm. c) Zoom in of dashed region in b) showing the movement of TCR micro-cluster as shown by the white arrow. Scale bar is 2 μm. d) Far left - Dual-colour kymograph showing the correlated motion of the actin cytoskeleton and TCR micro-clusters. Scale bar is 2 μm. Middle left – Single particle tracking of TCR micro-clusters during activation. Middle right – Quantification of TCR micro-cluster displacement. Far right - the distribution of TCR micro-cluster velocity with a mean of 85.5 ± 31.9 nm/s. e) Dual colour FRAP of actin (green) and plasma membrane (red) as labelled by SNAP-Cell-505 and CellMask Deep Red respectively during T-cell activation. Right – time-lapse imaging showing the recovery of the bleach region of actin (green) and plasma membrane (red). Scale bar is 2 μm. f) Quantification of the FRAP intensity recovery for actin and plasma membrane, highlighting the contrasting dynamics. g) Diffusion coefficient of the plasma membrane in the lamellipodium of an activating T-cell for the membrane dye CellMask Deep Red and labelled cholesterol (Chol-PEG-KK114) as measured by FCS. h) Kymograph analysis of actin and membrane dynamics showing correlative dynamics. Scale bar is 1 μm.
