# Supplementary material for: Cytoskeletal Control of Antigen-Dependent T Cell Activation
Source: Cell Rep. 2019 Mar 19;26(12):3369–3379.e5. doi: 10.1016/j.celrep.2019.02.074 (PMC6426652; doi:10.1016/j.celrep.2019.02.074)
Supplement: Document S2. Article plus Supplemental Information [file mmc9.pdf]

Cell Reports

Article

Cytoskeletal Control of Antigen-Dependent T Cell Activation

Graphical Abstract

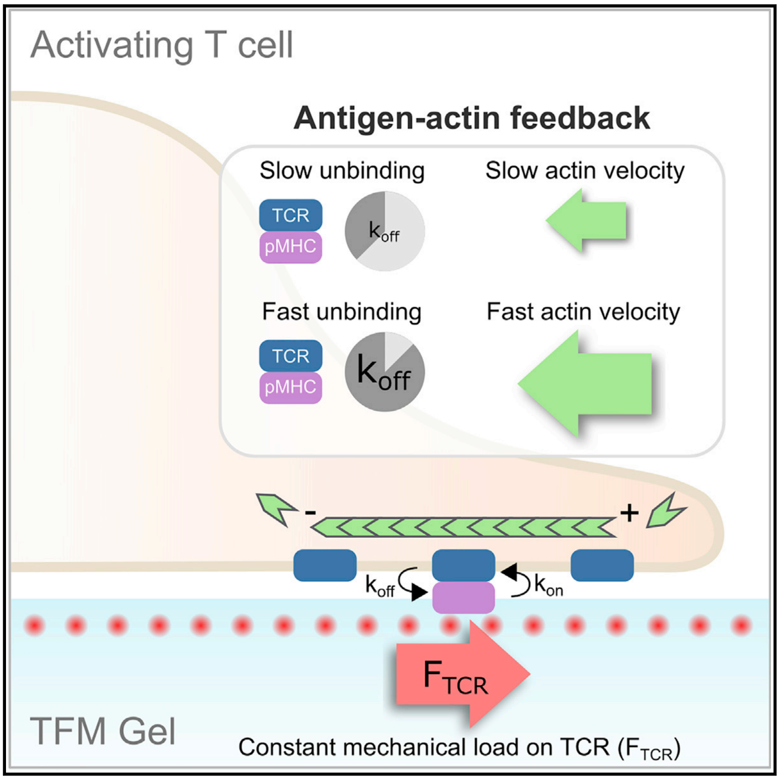

Highlights

- Mechanical force experienced by the TCR at the immune synapse is actin dependent
- Peak forces experienced by the TCR are independent of the antigen unbinding kinetics
- Actin velocity at the immune synapse is dependent on the stimulating antigen

Authors

Huw Colin-York, Yousef Javanmardi, Mark Skamrahl, ..., Vincenzo Cerundolo, Christian Eggeling, Marco Fritzsche

Correspondence

marco.fritzsche@rdm.ox.ac.uk

In Brief

T cell activation relies on a dynamic actin cytoskeleton. Here, Colin-York et al. show how the kinetics of the stimulating antigen influence the dynamics of actin. This feedback mechanism influences the mechanics at the immune synapse, allowing T cells to orchestrate the length scale and timescale of signaling.

Colin-York et al., 2019, Cell Reports *26*, 3369–3379
March 19, 2019 © 2019 The Author(s).
https://doi.org/10.1016/j.celrep.2019.02.074

CellPress

OPEN ACCESS
CellPress

Cytoskeletal Control of Antigen-Dependent T Cell Activation

Huw Colin-York,[1] Yousef Javanmardi,[2] Mark Skamrahl,[1] Sudha Kumari,[3] Veronica T. Chang,[4] Satya Khuon,[5] Aaron Taylor,[5] Teng-Leong Chew,[5] Eric Betzig,[5] Emad Moeendarbary,[2,6] Vincenzo Cerundolo,[1] Christian Eggeling,[1] and Marco Fritzsche[1,7,8,*]
[1]MRC Human Immunology Unit, Weatherall Institute of Molecular Medicine, University of Oxford, Headley Way, Oxford OX3 9DS, UK
[2]Department of Mechanical Engineering, University College London, London WC1E 7JE, UK
[3]Koch Institute of Integrative Cancer Research, Massachusetts Institute of Technology, Cambridge, MA 02139, USA
[4]MRC Laboratory of Molecular Biology, Francis Crick Avenue, Cambridge Biomedical Campus, Cambridge CB2 0QH, UK
[5]Janelia Research Campus, Howard Hughes Medical Institute, 19700 Helix Drive, Ashburn, VA 20147, USA
[6]Department of Biological Engineering, Massachusetts Institute of Technology, Cambridge, MA 02139, USA
[7]Kennedy Institute for Rheumatology, University of Oxford, Roosevelt Drive, Oxford OX3 7LF, UK
[8]Lead Contact
*Correspondence: marco.fritzsche@rdm.ox.ac.uk
https://doi.org/10.1016/j.celrep.2019.02.074

SUMMARY

Cytoskeletal actin dynamics is essential for T cell activation. Here, we show evidence that the binding kinetics of the antigen engaging the T cell receptor influences the nanoscale actin organization and mechanics of the immune synapse. Using an engineered T cell system expressing a specific T cell receptor and stimulated by a range of antigens, we found that the peak force experienced by the T cell receptor during activation was independent of the unbinding kinetics of the stimulating antigen. Conversely, quantification of the actin retrograde flow velocity at the synapse revealed a striking dependence on the antigen unbinding kinetics. These findings suggest that the dynamics of the actin cytoskeleton actively adjusted to normalize the force experienced by the T cell receptor in an antigen-specific manner. Consequently, tuning actin dynamics in response to antigen kinetics may thus be a mechanism that allows T cells to adjust the lengthscale and timescale of T cell receptor signaling.

INTRODUCTION

Cells adapt their biomechanics to fulfill their function in a range of complex, physical environments. During T cell activation, rearrangements of the actin cytoskeleton lead to mechanical changes within the cell, driven by the nanoscale organization of individual actin filaments (Fritzsche et al., 2017; Huse, 2017). It is now emerging that cells can dynamically regulate their mechanics to meet their physiological needs via a diverse range of feedback mechanisms (Elosegui-Artola et al., 2016; Roca-Cusachs et al., 2017; Schwarz and Gardel, 2012). In this way, external stimuli may lead to mechanical transitions within the cell, which consequently influence a functional outcome, such as the effector function of T cells. Molecular interactions at the T cell membrane initiate activation, but what follows is likely to involve a complex balance between the kinetics of key receptor-ligand interactions, the dynamics of the actin cytoskeleton, and the level of mechanical force generation. Understanding the nature of feedback between these components is of critical importance in providing a more complete understanding of T cell activation.

T cell receptor (TCR) binding to the peptide-loaded major histocompatibility complex (pMHC) on the surface of the antigen-presenting cell (APC) results in an actin-driven morphological change of the T cell. This change ultimately culminates in the formation of an organized cell-cell contact between the T cell and the APC, known as the immune synapse (IS). In the early stages of activation, T cells spread over the surface of the APC via the formation of a filamentous actin (F-actin)-rich lamellipodium, similar to that observed at the leading edge of a migrating cell (Yam et al., 2007). This morphological change occurs as a direct result of signaling downstream of TCR phosphorylation, where activation of the actin effector proteins Wiskott-Aldrich syndrome protein (WASp) and WASp family verprolin-homologous protein (WAVE) leads to the activation of the actin nucleator Arp2/3 (Fritzsche et al., 2017; Kumari et al., 2014). By binding existing actin filaments, the Arp2/3 complex results in the polymerization of short-branched actin filaments, accounting for the majority of the F-actin within the lamellipodium (Fritzsche et al., 2013, 2016). In addition to Arp2/3-mediated polymerization, formin binding leads to the extension of existing long actin filaments, which coupled with myosin-II contractility yields the formation of actin arcs (Murugesan et al., 2016). After the initial spreading phase, the contact area between the T cell and the APC stabilizes, and actin undergoes robust retrograde flow directed toward the center of the IS. Simultaneously, TCR micro-clusters form at the periphery of the IS and migrate inward as sites of active signaling in an actin-dependent manner (Dustin et al., 2007; Kaizuka et al., 2007). TCR signaling is concentrated in this outer region, correlating with the region of the most dynamic actin. Actin stabilization by pharmacological perturbation results in arrested TCR migration and a drop in intracellular

This is an open access article under the CC BY license (http://creativecommons.org/licenses/by/4.0/).

Ca^{2+} levels, indicating that a dynamic actin cytoskeleton is necessary to sustain the level of signaling via the TCR (Babich and Burkhardt, 2013; Babich et al., 2012).

The active movement of TCR clusters necessitates the generation of mechanical force at the IS, and together with the kinetic parameters of the stimulating antigens, it is likely to be key in determining the length scale and timescale of signaling via the TCR (Aleksic et al., 2010). Consequently, the duration of individual TCR clusters engaging MHCs or the distance traveled defines a characteristic signaling timescale or length scale, respectively. The kinetic parameters of the stimulating antigen have been shown to correlate with the strength of the T cell response, particularly the off rate of the binding kinetics (k_{off}) of the TCR-pMHC interaction (Aleksic et al., 2010). Because the k_{off} is a measure of the timescale of the TCR-pMHC interaction, in combination with the dynamics of the actin cytoskeleton, it also is critical in determining the overall mechanics at the IS. Moreover, a number of studies have demonstrated that the TCR itself may be mechanosensitive. Experiments using optical traps (Das et al., 2015), biomembrane force probes (Liu et al., 2014), and atomic force microscopy (AFM) (Hu and Butte, 2016) have demonstrated that applying tension to the TCR-pMHC interaction can enhance TCR-specific signaling. In support of these observations, the TCR-pMHC has been shown to exhibit slip-catch bond behavior, whereby forces applied to the interaction modify the kinetics, suggesting that T cells may discriminate antigens using mechanical force (Klotzsch and Schütz, 2013; Sibener et al., 2018). Despite this, little is understood of how such mechanosensitivity may be controlled at the IS. Only recently has it been shown how actin arcs generated by myosin-II-mediated contractility may serve to generate the necessary forces to promote activation via a mechanosensitive mechanism (Hong et al., 2017).

Therefore, what is lacking from our current understanding is how the rearrangement of the T cell cytoskeleton initiated by early signaling events influences those at later time points due to the changing mechanical environment in which TCR trafficking and signaling persist. The correlated motion of TCR micro-clusters and actin flow at the IS indicates that the two systems are tightly coupled (Smoligovets et al., 2012; Yu et al., 2010). In addition, traction force microscopy (TFM) has demonstrated mechanical force production during the formation and maintenance of the IS (Bashour and Gondarenko, 2014; Hui et al., 2015). However, mechanistic insights into how forces are transmitted to and experienced by the TCR from the actin cytoskeleton remain elusive. More generally, it is unknown whether the actin cytoskeleton serves simply as a platform for signaling or whether the dynamics of the actin cytoskeleton is playing an active role by tuning its dynamics to control the parameters of the TCR-pMHC interaction. In support of an active role for the cytoskeleton, we recently demonstrated how T cells form a ramified actin network beneath the IS, which adjusted its mechanics to facilitate activation (Fritzsche et al., 2017). Consistent with this, active force production by T cells has been shown to promote the stability of the IS between T cells and APCs through the enhanced activation of the integrin lymphocyte function-associated antigen 1 (LFA-1), coupled with the hindered mobility of intercellular adhesion molecule 1 (ICAM-1) on the surface of the APC (Comrie et al., 2015). Consequently, it is of critical importance to investigate whether feedback exists between the kinetics of the stimulating antigen and the dynamics of the actin cytoskeleton, which may offer a mechanism by which the T cell is able to control molecular events at the IS via moderation of the level of mechanical force generation (Thauland et al., 2017).

Here, using Jurkat T cells expressing the New York esophageal squamous cell carcinoma 1 (NY-ESO-1)$_{157–165}$-specific 1G4-TCR (Chen et al., 2005), we directly measure the mechanical forces experienced by the TCR at the IS by traction force experiments, and firmly establish how actin dynamics is influenced by the kinetics of the stimulating antigen. Combining a range of biophysical tools, we highlight a mechanism by which the T cell is able to influence activation, tuning the dynamics of cortical actin architecture to each specific antigen to normalize the level of mechanical force experienced by the TCR. These findings suggest that modulating actin dynamics in response to antigen kinetics may be a mechanism that allows T cells to adjust the lengthscale and timescale of TCR signaling.

RESULTS

Activating Jurkat T Cells Generate Mechanical Force Whose Magnitude Is Independent of Antigen Kinetics

To investigate the mechanical force experienced by TCRs during T cell activation, we used TFM. The elastic 3 kPa polyacrylamide (PAA) gel surface necessary for TFM was loaded with fluorescent beads and functionalized with histocompatibility leukocyte antigen (HLA) A2 molecules loaded with the NY-ESO-1$_{157–165}$ peptide (pMHC) recognized by the 1G4-TCR expressed by the Jurkat T cells (Figures 1A and S1). A stiffness of 3 kPa was chosen as a representative soft surface similar to that present during the physiological T cell-APC interaction, as well as being stiff enough to maintain a linear regime of mechanical force during TFM measurements (Bufi et al., 2015; Saitakis et al., 2017). The high-affinity peptide NY-ESO-1$_{157–165}$ peptide analog 9V (containing cysteine-to-valine substitution at position 165) was used for these experiments (Chen et al., 2005). Using TFM in combination with Jurkat T cells expressing fluorescently tagged monomeric actin (see Method Details), we could monitor the dynamics of the actin cytoskeleton and traction force generation simultaneously. As expected, on coming into contact with the gel surface, the Jurkat T cells rapidly spread over the surface, forming a stable contact characterized by an actin-rich lamellipodium at the periphery (Figure 1B), as has been previously shown on functionalized glass surfaces and supported lipid bilayers (SLBs) (Dustin et al., 2007).

By analyzing the displacement of the beads below the activating T cell, it was evident that the majority of mechanical force was concentrated at the periphery of the cell contact, with a large number of beads being displaced in this region compared to the center of the contact. Contrary to the expectation of widespread and continuous force generation, the nature of the observed forces was localized and displayed a distinct pattern. Under the lamellipodium, the bead motion was characteristic of so-called load-fail events (Chan and Odde, 2008). As is evident from the kymograph and bead tracking analysis, the beads

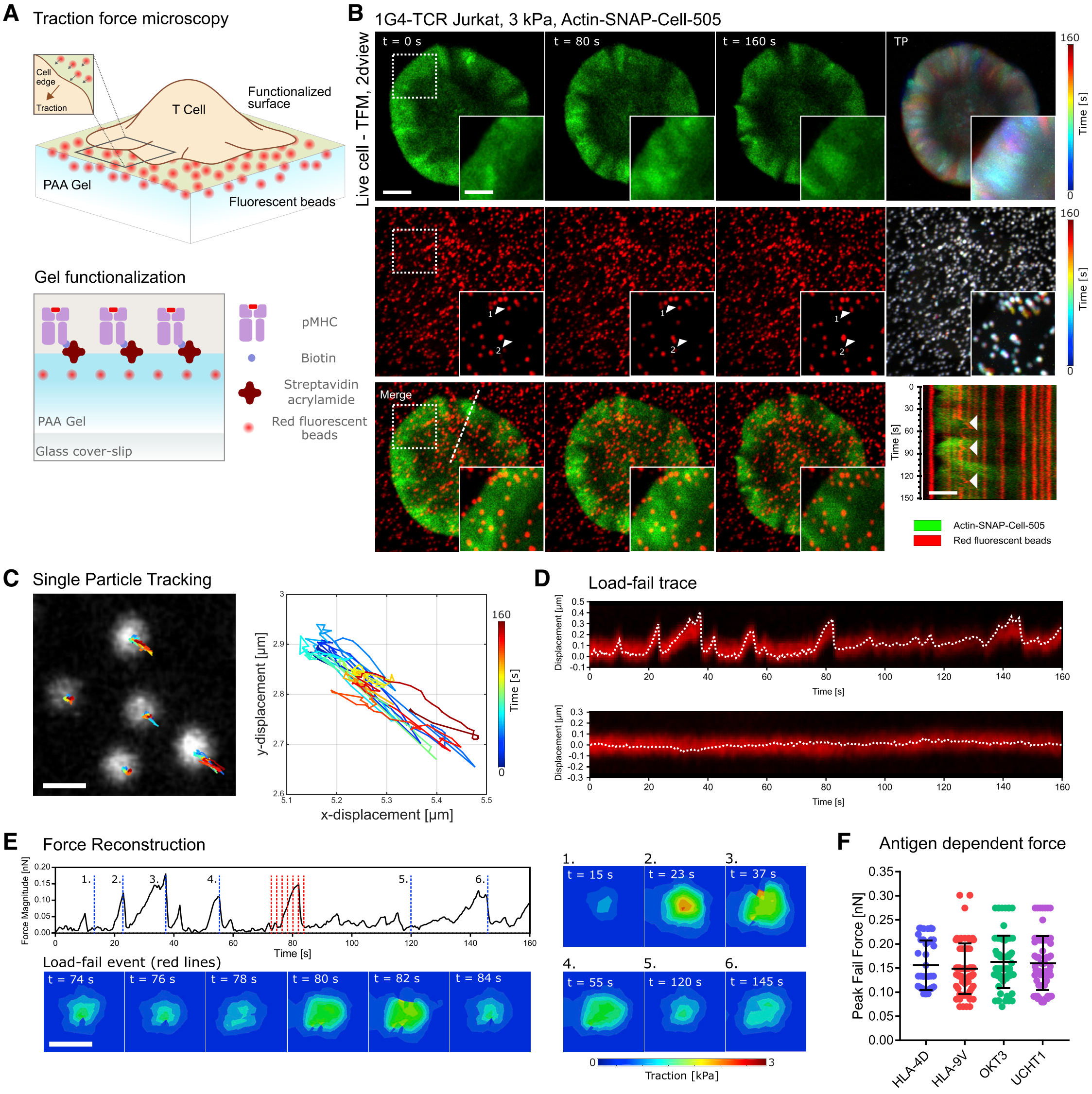

Figure 1. TFM Revealed Localized Forces during T Cell Activation Whose Peak Force Was Invariant to the Kinetics of the Stimulating Antigen

(A) Schematic of the TFM experimental setup, showing fluorescent beads localized to the top surface of 20- to 30-μm-thick PAA gel containing streptavidin-acrylamide and the upper surface functionalized as shown using biotinylated pMHC or anti-CD3 antibodies.

(B) Confocal fluorescent imaging time-lapse of a 1G4-TCR Jurkat T cell expressing actin-SNAP and labeled with SNAP-Cell-505 (green) interacting with a 3-kPa PAA gel functionalized with HLA-9V pMHC molecules and loaded with 40 nm red fluorescent beads. Scale bar, 2 μm. Inset shows a close-up of the dashed boxes. Scale bar, 1 μm. Top and center right show the temporal projection of both actin and bead dynamics. Bottom right shows a dual-color kymograph of a radial line profile of the activating T cell. Bead motion is visible in the outer lamellipodial region, whereas little bead motion is observed in the center of the cell contact.

(C) Left: single particle tracking of a representative selection of beads showing unidirectional bead motion. Scale bar, 0.5 μm. Right: close-up image of the individual bead track, showing color-coded temporal evolution of bead motion.

(D) Top: kymograph with projected track overlay, showing the characteristic load-fail dynamics of the bead displacement. Cyclical events characterized by a slow loading phase and a rapid return to an equilibrium position are observed in beads in the outer part of the cell contact. Bottom: representative kymograph of bead displacement in a region under the center of the cell contact, showing the absences of any directed motion, which indicates a lack of traction forces.

(E) Finite element (FE) analysis of a representative load-fail event, showing the evolution of force on the gel extracted from the displacement of the bead in (D). The evolution of force during a single load-fail event is indicated by the spatial distribution of forces shown in the heatmaps, with the corresponding time points indicated by the colored dashed line on the time course. Scale bar, 0.5 μm.

(F) Distribution of peak fail forces as calculated by finite element analysis for each antigen stimulation condition. Error bars show means and SDs; N ≥ 39 load-fail events, 10 cells per condition.

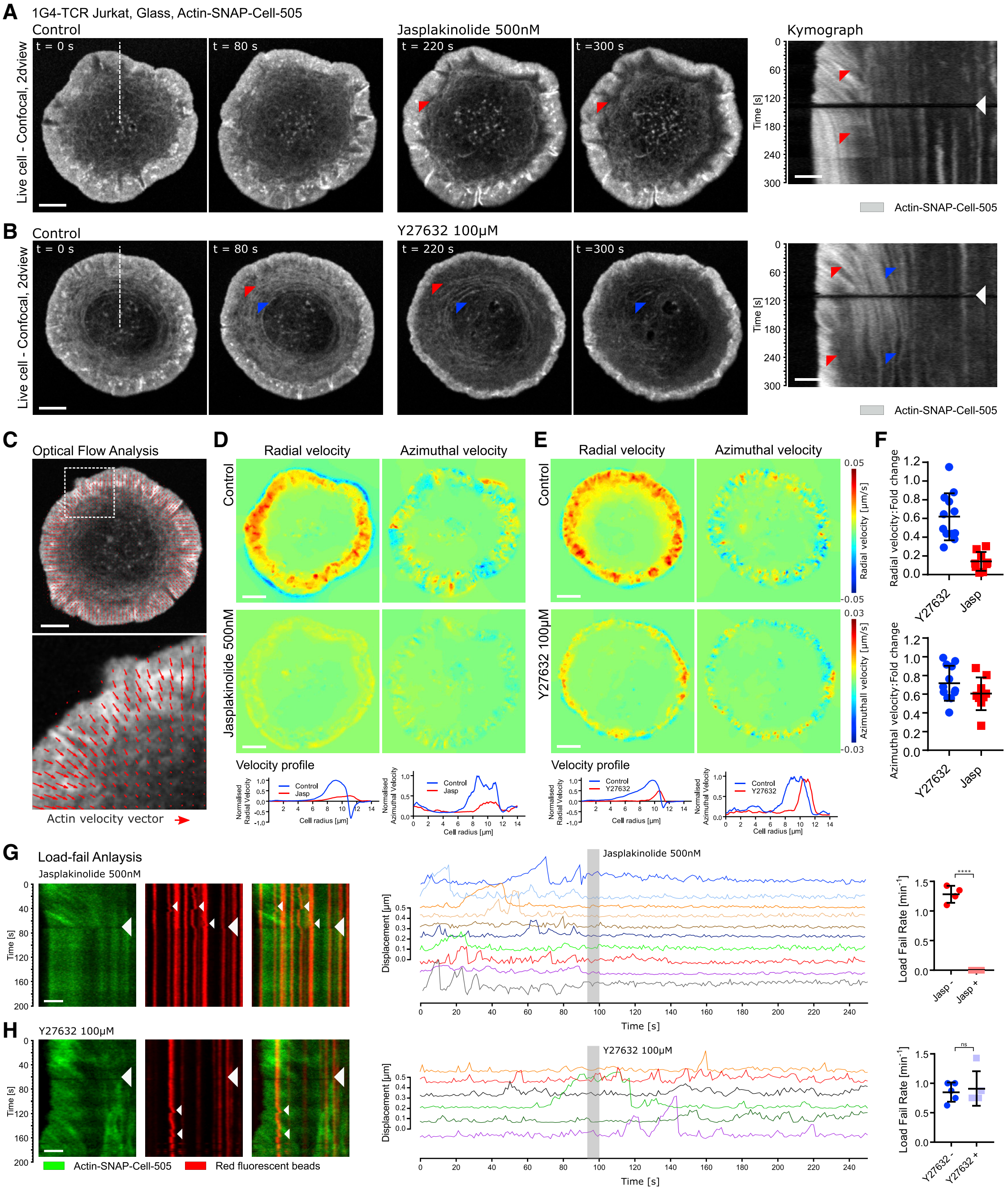

Figure 2. Load-Fail Dynamics Is Dependent on Actin Dynamics but Not Myosin-II Contractility

(A) Confocal fluorescent imaging time-lapse of a 1G4-TCR Jurkat T cell expressing actin-SNAP labeled with SNAP-Cell-505 interacting with an antigen-coated coverslip before and after the addition of 500 nM jasplakinolide. The red arrowheads indicate stationary features in the lamellipodium after treatment. At right is the corresponding kymograph. The red arrowheads show actin retrograde flow before treatment and arrested flow after treatment.

(B) Confocal fluorescent imaging time-lapse of a 1G4-TCR Jurkat T cell expressing actin-SNAP labeled with SNAP-Cell-505 interacting with an antigen-coated coverslip before and after the addition of 100 μM Y27632. The red arrowheads indicate lamellipodial width reduction after treatment. The blue arrowheads indicate the loss of actin arcs following treatment. At right is the corresponding kymograph.

(C) Optical flow analysis of actin flow during activation. The red arrows indicate a vector field, showing the direction of actin flow. Scale bar, 2 μm. At bottom is an extreme closeup of the dashed box at top.

(D) Pseudo-color intensity map of radial and azimuthal actin velocity before and after the addition of 500 nM jasplakinolide. Scale bar, 2 μm. Bottom: corresponding line profiles.

(E) Pseudo-color intensity map of radial and azimuthal actin velocity before and after the addition of 100 μM Y27632. Scale bar, 2 μm. Bottom: corresponding line profiles.

(legend continued on next page)

were initially displaced in a direction that correlated with the actin flow, toward the center of the IS (loading phase) (Figures 1C and 1D). The displacement increased gradually before reaching a maximum, at which point the bead appeared to return elastically to its equilibrium position (failing event). These load-fail events would often repeat, with multiple cycles of loading and failing during the activation period of 3 min, and each load event persisted for a median of 9.3 ± 6.5 s and traveled a median length of 139 ± 50 nm before failing (Video S1). Because surface functionalization was achieved using the specific pMHC alone, it follows that the forces observed were solely a consequence of TCR-pMHC contacts linking the cell and gel substrate, and hence serve as a measure of the forces directly experienced by the TCR. To further highlight that the forces were specific to the TCR-pMHC complexes, we functionalized the gel simultaneously with poly-L-lysine (PLL), as has been done during previous studies of force generation during T cell activation (Hui et al., 2015). The observed forces were strikingly different (Figure S2; Video S2), with global contractile forces gradually increasing over the period of 10 min. Notably, these differences were in line with recent work showing that PLL strongly activates T cells (Santos et al., 2018). These observations suggest that the adhesive contact of the PLL masked the localized load-fail forces, indicating a possible reason why they have not been previously observed in T cells.

The kinetic parameters of the antigen are key determinants of the T cell activation response. Mechanistically, antigens having a lower k_{off} would be bound to the TCR for a longer period. Hence, assuming a constant actin flow rate, this would result in a larger distance traveled by the TCR while still bound to the pMHC compared to the antigen with higher k_{off}. Assuming a homogeneous and elastic opposing surface, one would therefore expect that antigens binding the TCR with a lower k_{off} would result in a greater mechanical load imposed by the actin retrograde flow. To investigate this model, TFM experiments were conducted on a gel functionalized with antigens of a differing k_{off}. Load-fail behavior was observed across all of the antigens and was qualitatively similar for both anti-CD3 antibodies (OKT3 and UCHT1) and pMHCs (HLA-A2 4D and HLA-A2 9V) of differing k_{off} (Aleksic et al., 2010; Chen et al., 2005; Kjer-Nielsen et al., 2004; Lee et al., 2017; Salmerón et al., 1991). Calculating the precise level of mechanical load for each antigen during such events was not possible using conventional TFM approaches and required numerical solving using finite element (FE) analysis (see Method Details) because of the localized nature of the forces. Force reconstruction resulted in a trace of the force generated in the vicinity of each bead over time, allowing the peak force experienced during each load-fail event in that region of the gel to be quantified (Figure 1E). As can be seen from Figure 1F, the mean peak force experienced by the TCR for each antigen condition was not significantly different ($p > 0.05$, one-way ANOVA test), and there was also no apparent trend (HLA-4D: 0.16 ± 0.05 nN, HLA-9V: 0.15 ± 0.05 nN, OKT3: 0.16 ± 0.05 nN, UCHT1: 0.16 ± 0.06 nN, means and SDs, $N \geq 39$ load-fail events, 10 cells per condition). Given the model outlined above, this is a surprising result, as stronger binding antigens would be expected to show a higher peak force on a homogeneous elastic substrate. Conversely, this result suggests that the level of mechanical force experienced by the TCR is modulated, depending on the kinetics of the antigen.

Load-Fail Dynamics Is Dependent on Actin Dynamics but Not Myosin-II Contractility

To gain an understanding of the mechanism responsible for the modulation of mechanical forces, it was vital to uncover the molecular processes governing the load-fail behavior observed during IS formation using specific cytoskeletal perturbations (Babich et al., 2012; Yi et al., 2012). We carefully characterized the effect of stabilizing actin filaments and inhibiting myosin-II contractility on the actin dynamics at the IS. Using this characterization, we then applied the same perturbations in combination with the force measurements to assess whether actin polymerization or myosin-II contractility was primarily responsible for force generation.

Jasplakinolide is a specific pharmacological agent that serves to stabilize actin filaments, and has previously been shown to arrest TCR micro-cluster trafficking (Babich et al., 2012). To assess the effects of actin stabilization on the overall cytoskeletal organization, 500 nM jasplakinolide was added to T cells expressing monomeric fluorescent actin after allowing a stable synapse to form on antigen-coated glass coverslips (Figure 2A). As can be seen from time-lapse fluorescent imaging and corresponding kymograph analysis, the addition of jasplakinolide resulted in the rapid stabilization of lamellipodial actin, leading to a dramatic slowdown of the actin flow.

Myosin-II contractility has been implicated in governing the spatial-temporal dynamics of the T cell synapse and in controlling TCR cluster migration (Hammer and Burkhardt, 2013; Ilani et al., 2009; Kumari et al., 2012); however, it is unclear how myosin-II-mediated contractility contributes to the dynamics of actin within the lamellipodium. Y27632 is commonly used to inhibit myosin-II contractility by specifically targeting rho-associated protein kinase (ROCK). By repeating the actin stabilization experiments, but this time adding 100 μM Y27632, the contribution of myosin-II to actin dynamics was investigated. Unlike jasplakinolide, Y27632 did not stabilize actin, but the overall

(F) Fold change of radial (top) and azimuthal (bottom) actin velocity within the lamellipodium of a 1G4-TCR Jurkat T cell interacting with an antigen-coated coverslip after the addition of 500 nM jasplakinolide or 100 μM Y27632. Error bars show means and SDs; N = 12 cells per condition.

(G) Left: kymograph showing the dynamics of actin and fluorescent beads before and after the addition of 500 nM jasplakinolide (time point indicated by the large arrowhead). Actin dynamics shows clear retrograde flow pre-treatment and the stabilization of flow immediately afterward. Load-fail events are visible before treatment, as indicated by the small white arrowheads, and the events are dramatically reduced following treatment. Scale bar, 1 μm. Center: representative selection of tracks showing the dynamics of beads pre- and post-addition of 500 nM jasplakinolide. Treatment leads to the complete termination of load-fail events. Right: quantification of load-fail event rate before and after the addition of jasplakinolide. Error bars show means and SDs; N = 4 cells.

(H) Left: kymograph showing the dynamics of actin and fluorescent beads before and after the addition of 100 μM Y27632 (time point indicated by the large arrowhead). Scale bar, 1 μm. Center: representative selection of tracks showing the dynamics of beads pre- and post-addition of 100 μM Y27632. Right: quantification of load-fail event rate before and after the addition of Y27632. Error bars show means and SDs; N = 5 cells.

organization of the synapse was perturbed (Figure 2B). Notably, the lamellipodial region became less prominent and decreased in its spatial width. In addition, actin arcs present in the lamellum disassembled, leading to a decrease in actin density within the lamellum (Figure 2B).

To further quantify the changes in the actin dynamics in response to each perturbation, we next applied optical flow analysis to the fluorescent time-lapse imaging (Liu, 2009). While visual inspection indicated that the dominant actin flow was centripetal, closer examination revealed that the flow was spatially more complex, with a significant variation in flow speed and direction over the contact area. The spatial dependence of actin flow speed also correlated with structural differences in the actin organization from the lamellipodium, with fast-moving dense actin filaments, to sparser slow-moving filaments in the lamellum (Figure S3; Video S3). Optical flow allowed these more complex components to be extracted by separating the actin velocity into a radial component (toward the center of the contact) and an azimuthal component (tangential to the cell edge) across the cell contact (Figure 2C) (Liu, 2009). Figure 2D highlights the effects of 500 nM jasplakinolide on both the radial and azimuthal components of actin flow. Following the treatment, the optical flow pseudo-color velocity map shows that the radial velocity is almost completely abolished and that the azimuthal velocity is reduced. Line profiles further reveal this distinct change in actin dynamics following actin stabilization. Figure 2E displays the equivalent quantification after the addition of 100 μM Y27632. The thinning of the lamellipodium observed in the fluorescent time-lapse imaging (Figure 2B) is clearly visible in the pseudo-color velocity maps for both the azimuthal and radial components. The magnitudes of both radial and azimuthal velocities are similar before and after treatment with Y27632, suggesting that while the organization of actin at the IS is dependent on myosin-II contractility, the actin dynamics is not. Line profiles of the radial and azimuthal components revealed this distinct change in actin organization following inhibition of myosin-II contractility. The change in lamellipodial actin radial velocity for each perturbation was further quantified by plotting the fold change in radial velocity before and after treatment (Figure 2F), supporting the conclusions drawn from the representative cells in Figures 2D and 2E (N = 12 cells per condition).

Next, we applied the described perturbations during the TFM measurements on activating T cells. After allowing a stable synapse to form, the addition of jasplakinolide resulted in a significantly reduced retrograde flow of actin and load-fail events, as is evident from the kymograph and bead traces shown in Figure 2G for a representative cell (Video S4; $p < 0.5$, N = 4 cells). This effect was quantified by analyzing the load-fail event rate before and after the addition of the drug, in which the rate fell to zero immediately after the addition of jasplakinolide. Equivalent experiments were performed using Y27632 (Figure 2H). Myosin-II inhibition did not significantly change the load-fail event rate, as is evident from the quantification shown in Figure 2H (Video S5; $p > 0.5$, N = 5 cells). This result indicates that it is the retrograde flow of actin that is primarily responsible for the observed forces and gives further support to the concept that a tight coupling between the TCR and the actin cytoskeleton is responsible for the transmission of force from actin to the TCR during activation.

Actin Flow Velocity at the IS Is Antigen Dependent

The pharmacological perturbations of the actin cytoskeleton presented above indicated that actin polymerization was primarily responsible for the forces experienced by the TCR. In support of this, it is understood that TCR dynamics during activation correlates with the motion of the actin cytoskeleton (Figure S4) (Kaizuka et al., 2007; Murugesan et al., 2016) and that a coupling between TCR and the actin cytoskeleton is likely to be responsible for TCR cluster migration (Figure S4; Video S6) (Smoligovets et al., 2012; Yu et al., 2010). These observations indicate that the length- and timescale of the TCR-pMHC interaction may be controlled by the dynamics of the underlying actin cytoskeleton. As has been shown, the peak force experienced under varying antigens was consistent, implying that the T cell employs a mechanism to normalize this force at the IS. Owing to the apparent coupling between TCR and the actin cytoskeleton, as well as the force dependence on actin polymerization, any tuning of the forces experienced by the TCR are likely to result from changes in the dynamics of the actin cytoskeleton. To investigate whether regulating the dynamics of the actin cytoskeleton provides this mechanism, we next sought to systematically measure the actin flow velocity in response to antigens of varying kinetics.

To address this, Jurkat T cells expressing the 1G4-TCR were stimulated on glass surfaces functionalized with one of the four antigens used in the previously described TFM measurements, namely with two pMHCs (HLA-9V and HLA-4D) and two anti-CD3 antibodies (OKT3 and UCHT1), each differing in their k_{off} (Aleksic et al., 2010; Kjer-Nielsen et al., 2004; Salmerón et al., 1991). For each cell, the actin flow velocity was quantified. The gel used in the TFM experiments deform under an applied load, as was apparent from the load-fail data presented in Figure 1. Conversely, glass is a non-compliant substrate, and any forces generated by the cell are not efficiently transferred to the substrate. Therefore, to accurately measure the antigen dependence of actin retrograde flow, we performed the quantification of actin flow velocity on glass, removing any effects induced by the deformation of the substrate. Because of the complexity in the actin flow field outlined in Figure 2, actin flow quantification was carried out using a photobleaching approach. A circular region at the leading edge of the T cell lamellipodium was photobleached (Figure 3A; Video S7), and continued actin polymerization at the leading edge of the lamellipodium resulted in the displacement of the bleached region in the direction of actin polymerization. By tracking this bleached region, the absolute value of the actin flow velocity could be reliably extracted by applying a linear fit to the region displacement (Figures 3B and 3C).

The response of the activating cells fell into two distinct categories. Once activated, all of the cells were seen to spread; however, the stability of the leading edge of the cells differed greatly, as was previously evident from the optical flow analysis. In some cells, the leading edge remained stationary (Figure 3D), but in other cells this would either expand, increasing the cell contract area, or contract (Figure 3E). The retrograde flow of actin is a

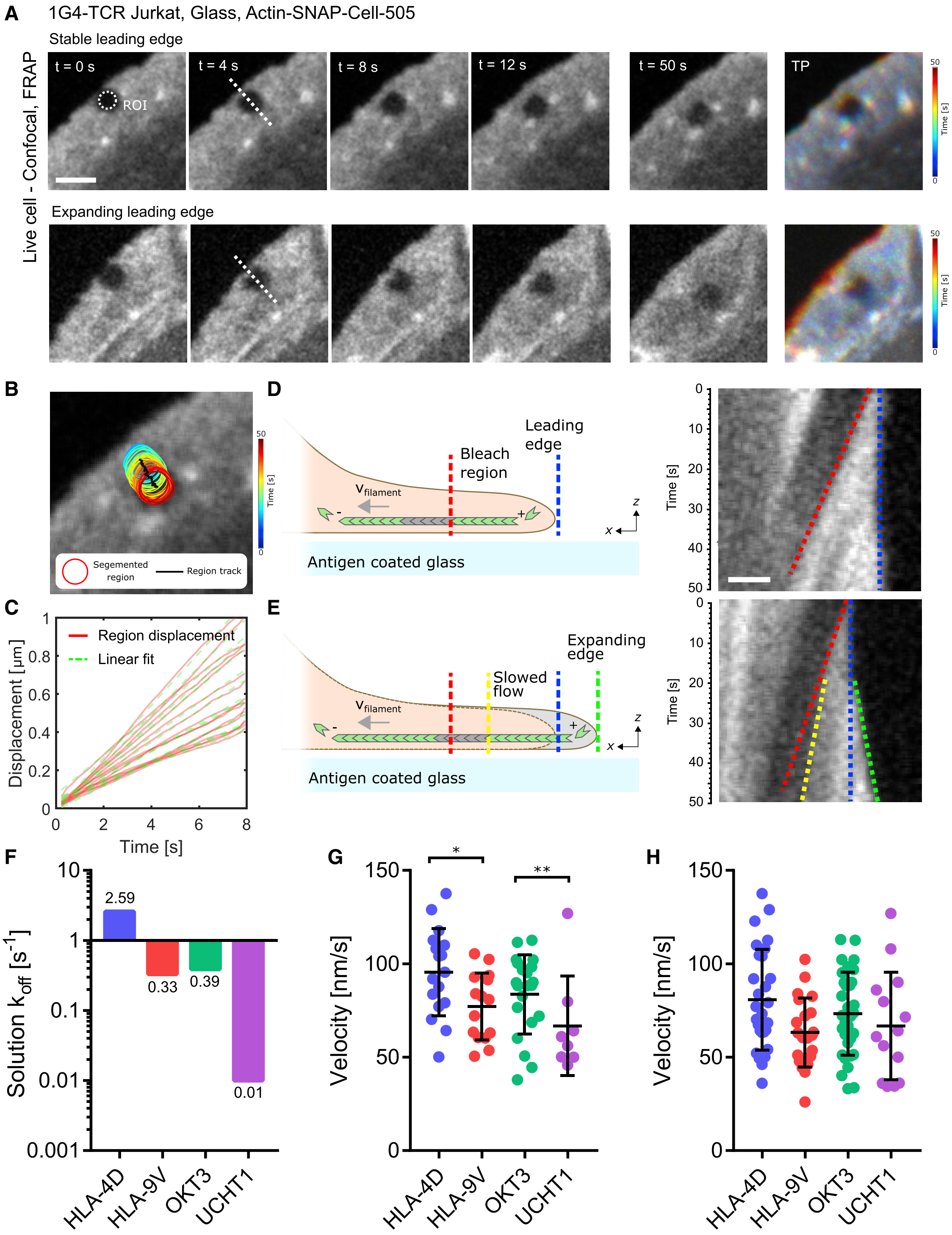

Figure 3. Actin Flow Velocity at the IS Is Antigen Dependent

(A) Time-lapse imaging for actin bleach region and temporal projection. Top: time-lapse shows a representative cell with a stable leading edge. Bottom: time-lapse shows a representative cell in which the leading edge expands over time. Scale bar, 1 μm.

(B) Tracking of segmented bleached region in the T cell lamellipodium.

(C) Displacement of segmented bleached region with corresponding linear fit.

(D) Left: model of actin bleach region dynamics in the presence of a stable leading edge. Right: kymograph of stable leading edge, showing the bleach region edge (red dotted line) and the leading edge (blue dotted line).

(legend continued on next page)

consequence of the actin filament polymerization rate and the tension of the plasma membrane (Mueller et al., 2017). In T cells in which the leading edge expands, the force of polymerization exceeds the membrane tension, and the converse is true in contracting cells. Because of this imbalance, the retrograde flow velocity in each of these cases would be affected. This observation is highlighted in Figures 3D and 3E; Figure 3D shows a kymograph of a stable leading edge and Figure 3E an expanding leading edge. Where the leading edge is stationary, the bleach region moves at a constant velocity, but conversely, when the leading edge expands, the bleach region decreases in velocity, suggesting a shift in balance between membrane tension and the force induced by actin polymerization (Mueller et al., 2017). By quantifying the actin flow velocity for cells exhibiting a stable leading edge for each antigen, it was possible to observe a distinct trend that reflected the k_{off} of the antigens (Figures 3F and 3G). The pMHC with the fastest $k_{off,}$ HLA-4D, showed the highest rate of actin flow, with a mean of 95.5 ± 23.3 nm/s, while the more strongly binding HLA-9V exhibited a significantly slower mean velocity of 77.12 ± 17.9 nm/s ($p < 0.05$, $N \geq 12$ cells per condition). The anti-CD3 antibodies consistently show a similar trend, with stimulation via the more strongly binding UCHT1 (66.73 ± 26.58 nm/s) resulting in a slower actin velocity compared to OKT3 (83.69 ± 21.14 nm/s) ($p < 0.05$, $N \geq 8$ cells per condition). If cells showing a non-stable leading edge were included in the quantification, then the trend was less distinct (Figure 3H), supporting the notion that changes in the membrane tension and the force induced by actin polymerization can alter the actin flow rate. This important result demonstrates that the kinetics of the antigen can affect the actin dynamics during activation, providing evidence in support of active feedback between antigen kinetics and actin dynamics.

DISCUSSION

Using a combination of biophysical tools, we have shown that cytoskeletal actin dynamics contributes to the control of T cell activation in an antigen-dependent manner. By adapting the velocity of actin retrograde flow, we speculate that the peak force experienced by the TCR is normalized across all antigens, suggesting a mechanism by which T cells may orchestrate the dynamics of the TCR during signaling at the IS. This result shows how the kinetic parameters of the antigen can influence the mechanics of the synapse, providing an important insight into how cells control their physiological function.

TFM using PAA gels functionalized with both pMHC and anti-CD3 antibodies exhibited highly localized and distinct force patterns, referred to as load-fail events. The peak mechanical force recorded in the presence of each of the four antigens was not significantly different, suggesting that T cells were able to adapt the level of force generation to each specific antigen. Mechanistically, load-fail forces were a consequence of actin retrograde flow, as highlighted by perturbation experiments, which is consistent with previous work (Hui et al., 2015). Stabilizing actin filaments pharmacologically resulted in the arrest of actin retrograde flow and a dramatic loss of force generation. Inhibition of myosin-II contractility, while affecting the overall actin organization, did not perturb the actin retrograde flow and had no significant effect on the observed force generation.

The invariance of force generation to antigens of differing kinetics is contrary to expectation, as strongly binding antigens would be expected to exhibit higher forces, owing to the increased lifetime of the TCR-pMHC interactions (Liu et al., 2016). However, this model relies on the assumption that the actin cytoskeleton provides a consistent level of mechanical force to induce TCR cluster migration, resulting from a constant actin flow velocity. The invariance of the peak force was therefore an indication that feedback between the antigen kinetics and the actin flow rate influenced the level of mechanical force generation. It is important to note that the mechanical forces measured here are unlikely to result from single TCR-antigen binding events, but rather clusters of molecules. Dissecting the effects of single TCR binding events from their collective binding remains challenging and could account for differences between our observations and previous work, in which an antigen dependence of force was measured (Liu et al., 2016). TFM revealed that the lengthscale and timescale of the load-fail events were approximately 150 nm and 10 s, respectively, indicating that the TCR would undergo multiple cycles of binding and unbinding during its transport across the IS. This indicates that TCR signaling is not continuous at any location and time at the lamellipodium, but rather that the actin cytoskeleton defines a spatial length- and timescale at which signaling is maintained at the cell surface. This highlights the importance of monitoring the cytoskeletal dynamics of actin in response to different antigens (Hong et al., 2017; Murugesan et al., 2016).

Load-fail force patterns like those observed here have previously been reported in neuronal growth cones and were interpreted as being as a result of mechanical forces generated by the actin cytoskeleton and transferred to the compliant substrate by temporary adhesions formed between the cell and the gel beneath (Chan and Odde, 2008). On forming an adhesion, the persistent flow of the actin cytoskeleton generates a force opposing the adhesive contact. As the force opposing the adhesion increases, the elastic substrate is gradually displaced. This increases up to a threshold where the force generated by the actin cytoskeleton outweighs the strength of the adhesive contact, at which point, the receptor-ligand interaction maintaining the adhesive contact will abruptly fail. This failing event is interpreted here as a passive process, but it could be actively

(E) Left: model of actin bleach region dynamics in the presence of an expanding leading edge. Right: kymograph of the expanding leading edge (blue and green dotted lines) and the bleached region edge, showing a corresponding slowdown as the leading edge expands (red and yellow dotted lines).
(F) Antigen kinetics as shown by their solution k_{off} for the four stimulating antigens.
(G) Distribution of actin velocity as measured by bleach region tracking for each antigen for cells exhibiting a stationary leading edge. Error bars show means and SDs; $N \geq 8$ cells per condition.
(H) Distribution of actin velocity as measured by bleach region tracking for each antigen for all activating cells (expanding, stationary, and contracting leading edges). Error bars show means and SDs; $N \geq 14$ cells per condition.

controlled by myosin motors (Feng et al., 2017). Higher spatial-temporal studies would be required to fully elucidate the nature of a fail event as an active or a passive process. This failing event could also emerge as a consequence of a rupture in the linkage between the receptor-ligand interaction maintaining the cell contact and the actin cytoskeleton. However, such a model in T cells requires either a specific interaction between actin and the TCR (e.g., via an adaptor protein) or a non-specific interaction (e.g., a frictional coupling) to transfer the forces generated by the cytoskeleton to the TCR on the membrane. While the force measurements presented here do not favor a specific or non-specific mechanism for this force transmission, the actin perturbation experiments indicate that actin flow is responsible for the observed forces. Furthermore, these measurements highlight the potential of TFM to provide an important readout that will likely be crucial in uncovering the full mechanism by which force transmission occurs between actin and the TCR (Ditlev et al., 2018). In addition, co-labeling of the TCR and actin during force generation would be beneficial in uncovering such a mechanism via a combination with advanced dynamic techniques; however, localization of TCR micro-clusters using confocal microscopy in combination with TFM would result in a large amount of intracellular TCR fluorescence signal, precluding the localization of TCR micro-clusters on the membrane (Schneider et al., 2018).

Finally, careful analysis of the actin retrograde flow velocity under varying antigen conditions showed a velocity dependence. This result builds upon previous work, in which it has been shown that the nano-scale organization and dynamics of the actin cytoskeleton changes between the resting and activated T cell (Fritzsche et al., 2017). Moreover, this result confirms a coupling between actin and the motion of the TCR, demonstrating feedback between the two systems. Notably, if the changes in actin velocity were a consequence of downstream signaling alone, one may expect that weak peptides would induce less actin polymerization, and hence a lower actin velocity, as the level of Ca^{2+} influx during activation has been shown to influence the retrograde flow rate (Hartzell et al., 2016). This is contrary to our observations, suggesting that a more complex and—crucially—physical feedback mechanism is at work. In support of this view, a recent study has shown that the elongation rate of actin filaments can be directly altered by the mechanical load applied to the filament, as could be the case in T cells, in which more strongly binding pMHC leads to an increased mechanical load and a slowdown in actin (Mueller et al., 2017). Given the invariance of the peak load force to the antigen kinetics, we speculate that feedback to the actin cytoskeleton via a direct coupling to the TCR serves to normalize this force. This result highlights a key mechanism by which the T cell could maintain robust signaling in response to a wide range of antigen stimulation by controlling the level of force experienced by the TCR micro-clusters, resulting in a constant length- and timescale of signaling via the TCR at the IS. Previous work has shown that the proportion of T cells showing a calcium response when stimulated by the same range of antigens as used here did not change, suggesting a robust signaling response (Lee et al., 2017). Despite this, differences were observed in the qualitative nature of the calcium flux, with weakly binding antigens tending to exhibit oscillatory responses compared to single peaked responses (Lee et al., 2017).

Our findings, while not directly in support, are consistent with a model that forces generated at the IS facilitate antigen discrimination via catch and slip bond behavior (Liu et al., 2014), whereby the apparent kinetics of the antigens measured in solution is modified by the mechanical forces generated at the IS. Moreover, our observations support a view that the generation of force that is most critical is the outer regions on the IS, correlating with the area of most active TCR signaling. To build upon this conclusion, future work should focus on uncovering the molecular details of the coupling between actin dynamics and the motion of the TCR micro-clusters—specifically, how actin is coupled to the TCR and whether the feedback observed here results from a passive or an active mechanism. In addition, the influence of other molecules that are key to synapse formation on the mechanical load generated during activation should be investigated. This insight will be critical in developing a complete understanding of how T cells are able to orchestrate events at the IS in response to differing antigens.

STAR★METHODS

Detailed methods are provided in the online version of this paper and include the following:

- KEY RESOURCES TABLE
- CONTACT FOR REAGENT AND RESOURCE SHARING
- EXPERIMENTAL MODEL AND SUBJECT DETAILS
- METHOD DETAILS
  - pMHC Production
  - Protein Biotinylation
  - Bilayer Preparation
- PAA GEL PREPARATION
  - PAA Gel Functionalisation
  - Coverslip Preparation
  - TFM data acquisition
  - TFM analysis
  - eTIRF-SIM microscopy
  - Optical flow analysis
  - TCR cluster tracking
  - FRAP and bleach region tracking
  - Fluorescence correlation spectroscopy
- QUANTIFICATION AND STATISTICAL ANALYSIS
  - Quantification
- GENERAL STATISTICAL METHODS

SUPPLEMENTAL INFORMATION

Supplemental Information can be found with this article online at https://doi.org/10.1016/j.celrep.2019.02.074.

ACKNOWLEDGMENTS

M.F. and H.C.-Y. thank the Wolfson Imaging Centre Oxford for providing microscope facility support, the Wellcome Trust (212343/Z/18/Z), and EPSRC (EP/S004459/1). The TIRF-SIM microscope was built in collaboration with and with funds from Micron (http://micronoxford.com), an Oxford-wide advanced microscopy technology consortium supported by Wellcome Strategic Awards (091911 and 107457) and an MRC/EPSRC/BBSRC next generation imaging award. Y.J. and E.M. are grateful for support from the Cancer Research UK Multidisciplinary Award (C57744/A22057) and Leverhulme Trust Research Project Grant (RPG-2018-443).

AUTHOR CONTRIBUTIONS

M.F. and H.C.-Y. developed the concept and designed the experimental approach. H.C.-Y., M.F., Y.J., and M.S. carried out the experiments and implemented the data analysis with the assistance of all of the authors. M.F. and H.C.-Y. wrote the article. S. Kumari, V.T.C., S. Khuon, A.T., T.-L.C., E.B., and V.C. supported the study with samples and experimental setups. All of the authors were involved in discussions of the data and manuscript editing.

DECLARATION OF INTERESTS

The authors declare no competing interests.

Received: December 11, 2017
Revised: August 6, 2018
Accepted: February 20, 2019
Published: March 19, 2019

REFERENCES

Aleksic, M., Dushek, O., Zhang, H., Shenderov, E., Chen, J.-L., Cerundolo, V., Coombs, D., and van der Merwe, P.A. (2010). Dependence of T cell antigen recognition on T cell receptor-peptide MHC confinement time. Immunity *32*, 163–174.

Babich, A., and Burkhardt, J.K. (2013). Coordinate control of cytoskeletal remodeling and calcium mobilization during T-cell activation. Immunol. Rev. *256*, 80–94.

Babich, A., Li, S., O'Connor, R.S., Milone, M.C., Freedman, B.D., and Burkhardt, J.K. (2012). F-actin polymerization and retrograde flow drive sustained PLCγ1 signaling during T cell activation. J. Cell Biol. *197*, 775–787.

Bashour, K., and Gondarenko, A. (2014). CD28 and CD3 have complementary roles in T-cell traction forces. Proc. Natl. Acad. Sci. USA *111*, 2241–2246.

Bufi, N., Saitakis, M., Dogniaux, S., Buschinger, O., Bohineust, A., Richert, A., Maurin, M., Hivroz, C., and Asnacios, A. (2015). Human Primary Immune Cells Exhibit Distinct Mechanical Properties that Are Modified by Inflammation. Biophys. J. *108*, 2181–2190.

Chan, C.E., and Odde, D.J. (2008). Traction dynamics of filopodia on compliant substrates. Science *322*, 1687–1691.

Chen, J.-L., Stewart-Jones, G., Bossi, G., Lissin, N.M., Wooldridge, L., Choi, E.M.L., Held, G., Dunbar, P.R., Esnouf, R.M., Sami, M., et al. (2005). Structural and kinetic basis for heightened immunogenicity of T cell vaccines. J. Exp. Med. *201*, 1243–1255.

Choudhuri, K., Llodrá, J., Roth, E.W., Tsai, J., Gordo, S., Wucherpfennig, K.W., Kam, L.C., Stokes, D.L., and Dustin, M.L. (2014). Polarized release of T-cell-receptor-enriched microvesicles at the immunological synapse. Nature *507*, 118–123.

Colin-York, H., Eggeling, C., and Fritzsche, M. (2017). Dissection of mechanical force in living cells by super-resolved traction force microscopy. Nat. Protoc. *12*, 783–796.

Comrie, W.A., Babich, A., and Burkhardt, J.K. (2015). F-actin flow drives affinity maturation and spatial organization of LFA-1 at the immunological synapse. J. Cell Biol. *208*, 475–491.

Das, D.K., Feng, Y., Mallis, R.J., Li, X., Keskin, D.B., Hussey, R.E., Brady, S.K., Wang, J.-H., Wagner, G., Reinherz, E.L., and Lang, M.J. (2015). Force-dependent transition in the T-cell receptor β-subunit allosterically regulates peptide discrimination and pMHC bond lifetime. Proc. Natl. Acad. Sci. USA *112*, 1517–1522.

Denisin, A.K., and Pruitt, B.L. (2016). Tuning the Range of Polyacrylamide Gel Stiffness for Mechanobiology Applications. ACS Appl. Mater. Interfaces *8*, 21893–21902.

Ditlev, J.A., Vega, A.R., Köster, D.V., Su, X., Lakoduk, A., Vale, R.D., Mayor, S., Jaqaman, K., and Rosen, M.K. (2018). A Composition-Dependent Molecular Clutch Between T Cell Signaling Clusters and Actin. bioRxiv. https://doi.org/10.1101/316414.

Dustin, M.L., Starr, T., Varma, R., and Thomas, V.K. (2007). Supported planar bilayers for study of the immunological synapse. Curr. Protoc. Immunol. *Chapter 18*, Unit 18.13.

Elosegui-Artola, A., Oria, R., Chen, Y., Kosmalska, A., Pérez-González, C., Castro, N., Zhu, C., Trepat, X., and Roca-Cusachs, P. (2016). Mechanical regulation of a molecular clutch defines force transmission and transduction in response to matrix rigidity. Nat. Cell Biol. *18*, 540–548.

Feng, Y., Brazin, K.N., Kobayashi, E., Mallis, R.J., Reinherz, E.L., and Lang, M.J. (2017). Mechanosensing drives acuity of $\alpha\beta$ T-cell recognition. Proc. Natl. Acad. Sci. USA *114*, E8204–E8213.

Fritzsche, M., and Charras, G. (2015). Dissecting protein reaction dynamics in living cells by fluorescence recovery after photobleaching. Nat. Protoc. *10*, 660–680.

Fritzsche, M., Lewalle, A., Duke, T., Kruse, K., and Charras, G. (2013). Analysis of turnover dynamics of the submembranous actin cortex. Mol. Biol. Cell *24*, 757–767.

Fritzsche, M., Erlenkämper, C., Moeendarbary, E., Charras, G., and Kruse, K. (2016). Actin kinetics shapes cortical network structure and mechanics. Sci. Adv. *2*, e1501337.

Fritzsche, M., Fernandes, R.A., Chang, V.T., Colin-York, H., Clausen, M.P., Felce, J.H., Galiani, S., Erlenkämper, C., Santos, A.M., Heddleston, J.M., et al. (2017). Cytoskeletal actin dynamics shape a ramifying actin network underpinning immunological synapse formation. Sci. Adv. *3*, e1603032.

Grakoui, A., Bromley, S.K., Sumen, C., Davis, M.M., Shaw, A.S., Allen, P.M., and Dustin, M.L. (1999). The Immunological Synapse: A Molecular Machine Controlling T Cell Activation. Science *285*, 221–227.

Hammer, J.A., 3rd, and Burkhardt, J.K. (2013). Controversy and consensus regarding myosin II function at the immunological synapse. Curr. Opin. Immunol. *25*, 300–306.

Hartzell, C.A., Jankowska, K.I., Burkhardt, J.K., and Lewis, R.S. (2016). Calcium influx through CRAC channels controls actin organization and dynamics at the immune synapse. eLife *5*, 35–46.

Hong, J., Murugesan, S., Betzig, E., and Hammer, J.A. (2017). Contractile actomyosin arcs promote the activation of primary mouse T cells in a ligand-dependent manner. PLoS One *12*, e0183174.

Honigmann, A., Mueller, V., Ta, H., Schoenle, A., Sezgin, E., Hell, S.W., and Eggeling, C. (2014). Scanning STED-FCS reveals spatiotemporal heterogeneity of lipid interaction in the plasma membrane of living cells. Nat. Commun. *5*, 5412.

Hu, K.H., and Butte, M.J. (2016). T cell activation requires force generation. J. Cell Biol. *213*, 535–542.

Hui, K.L., Balagopalan, L., Samelson, L.E., and Upadhyaya, A. (2015). Cytoskeletal forces during signaling activation in Jurkat T-cells. Mol. Biol. Cell *26*, 685–695.

Huse, M. (2017). Mechanical forces in the immune system. Nat. Rev. Immunol. *17*, 679–690.

Ilani, T., Vasiliver-Shamis, G., Vardhana, S., Bretscher, A., and Dustin, M.L. (2009). T cell antigen receptor signaling and immunological synapse stability require myosin IIA. Nat. Immunol. *10*, 531–539.

Kaizuka, Y., Douglass, A.D., Varma, R., Dustin, M.L., and Vale, R.D. (2007). Mechanisms for segregating T cell receptor and adhesion molecules during immunological synapse formation in Jurkat T cells. Proc. Natl. Acad. Sci. USA *104*, 20296–20301.

Kjer-Nielsen, L., Dunstone, M.A., Kostenko, L., Ely, L.K., Beddoe, T., Mifsud, N.A., Purcell, A.W., Brooks, A.G., McCluskey, J., and Rossjohn, J. (2004). Crystal structure of the human T cell receptor CD3 $\varepsilon\gamma$ heterodimer complexed to the therapeutic mAb OKT3. Proc. Natl. Acad. Sci. USA *101*, 7675–7680.

Klotzsch, E., and Schütz, G.J. (2013). Improved ligand discrimination by force-induced unbinding of the T cell receptor from peptide-MHC. Biophys. J. *104*, 1670–1675.

Kumari, S., Vardhana, S., Cammer, M., Curado, S., Santos, L., and Michael, P. (2012). T lymphocyte myosin IIA is required for maturation of the immunological synapse. Front. Immunol. *3*, 1–13.

Kumari, S., Curado, S., Mayya, V., and Dustin, M.L. (2014). T cell antigen receptor activation and actin cytoskeleton remodeling. Biochim. Biophys. Acta *1838*, 546–556.

Lee, A.M., Colin-York, H., and Fritzsche, M. (2017). CalQuo^2: automated Fourier-space, population-level quantification of global intracellular calcium responses. Sci. Rep. *7*, 5416.

Li, C., Xu, C., Gui, C., and Fox, M.D. (2010). Distance regularized level set evolution and its application to image segmentation. IEEE Trans. Image Process. *19*, 3243–3254.

Li, D., Shao, L., Chen, B.-C., Zhang, X., Zhang, M., Moses, B., Milkie, D.E., Beach, J.R., Hammer, J.A., 3rd, Pasham, M., et al. (2015). ADVANCED IMAGING. Extended-resolution structured illumination imaging of endocytic and cytoskeletal dynamics. Science *349*, aab3500.

Liu, C. (2009). Beyond Pixels: Exploring New Representations and Applications for Motion Analysis (Massachusetts Institute of Technology).

Liu, B., Chen, W., Evavold, B.D.D., and Zhu, C. (2014). Accumulation of dynamic catch bonds between TCR and agonist peptide-MHC triggers T cell signaling. Cell *157*, 357–368.

Liu, Y., Blanchfield, L., Ma, V.P.-Y., Andargachew, R., Galior, K., Liu, Z., Evavold, B., and Salaita, K. (2016). DNA-based nanoparticle tension sensors reveal that T-cell receptors transmit defined pN forces to their antigens for enhanced fidelity. Proc. Natl. Acad. Sci. USA *113*, 5610–5615.

Mueller, J., Szep, G., Nemethova, M., de Vries, I., Lieber, A.D., Winkler, C., Kruse, K., Small, J.V., Schmeiser, C., Keren, K., et al. (2017). Load Adaptation of Lamellipodial Actin Networks. Cell *171*, 188–200.e16.

Murugesan, S., Hong, J., Yi, J., Li, D., Beach, J.R., Shao, L., Meinhardt, J., Madison, G., Wu, X., Betzig, E., and Hammer, J.A. (2016). Formin-generated actomyosin arcs propel T cell receptor microcluster movement at the immune synapse. J. Cell Biol. *215*, 383–399.

Roca-Cusachs, P., Conte, V., and Trepat, X. (2017). Quantifying forces in cell biology. Nat. Cell Biol. *19*, 742–751.

Saitakis, M., Dogniaux, S., Goudot, C., Bufi, N., Asnacios, S., Maurin, M., Randriamampita, C., Asnacios, A., and Hivroz, C. (2017). Different TCR-induced T lymphocyte responses are potentiated by stiffness with variable sensitivity. eLife *6*, e23190.

Salmerón, A., Sánchez-Madrid, F., Ursa, M.A., Fresno, M., and Alarcón, B. (1991). A conformational epitope expressed upon association of CD3-epsilon with either CD3-delta or CD3-gamma is the main target for recognition by anti-CD3 monoclonal antibodies. J. Immunol. *147*, 3047–3052.

Santos, A.M., Ponjavic, A., Fritzsche, M., Fernandes, R.A., de la Serna, J.B., Wilcock, M.J., Schneider, F., Urbančič, I., McColl, J., Anzilotti, C., et al. (2018). Capturing resting T cells: the perils of PLL. Nat. Immunol. *19*, 203–205.

Schneider, F., Waithe, D., Lagerholm, B.C., Shrestha, D., Sezgin, E., Eggeling, C., and Fritzsche, M. (2018). Statistical Analysis of Scanning Fluorescence Correlation Spectroscopy Data Differentiates Free from Hindered Diffusion. ACS Nano *12*, 8540–8546.

Schwarz, U.S., and Gardel, M.L. (2012). United we stand: integrating the actin cytoskeleton and cell-matrix adhesions in cellular mechanotransduction. J. Cell Sci. *125*, 3051–3060.

Sibener, L.V., Fernandes, R.A., Kolawole, E.M., Carbone, C.B., Liu, F., McAffee, D., Birnbaum, M.E., Yang, X., Su, L.F., Yu, W., et al. (2018). Isolation of a Structural Mechanism for Uncoupling T Cell Receptor Signaling from Peptide-MHC Binding. Cell *174*, 672–687.e27.

Smoligovets, A.A., Smith, A.W., Wu, H.-J., Petit, R.S., and Groves, J.T. (2012). Characterization of dynamic actin associations with T-cell receptor microclusters in primary T cells. J. Cell Sci. *125*, 735–742.

Thauland, T.J., Hu, K.H., Bruce, M.A., and Butte, M.J. (2017). Cytoskeletal adaptivity regulates T cell receptor signaling. Sci. Signal. *10*, eaah3737.

Tse, J.R., Engler, A.J., Tse, J.R., and Engler, A.J. (2010). Preparation of hydrogel substrates with tunable mechanical properties. Curr. Protoc. Cell Biol. *Chapter 10*, Unit 10.16.

Waithe, D., Clausen, M.P., Sezgin, E., and Eggeling, C. (2016). FoCuS-point: software for STED fluorescence correlation and time-gated single photon counting. Bioinformatics *32*, 958–960.

Yam, P.T., Wilson, C.A., Ji, L., Hebert, B., Barnhart, E.L., Dye, N.A., Wiseman, P.W., Danuser, G., and Theriot, J.A. (2007). Actin-myosin network reorganization breaks symmetry at the cell rear to spontaneously initiate polarized cell motility. J. Cell Biol. *178*, 1207–1221.

Yi, J., Wu, X.S., Crites, T., and Hammer, J.A., 3rd. (2012). Actin retrograde flow and actomyosin II arc contraction drive receptor cluster dynamics at the immunological synapse in Jurkat T cells. Mol. Biol. Cell *23*, 834–852.

Yu, C.H., Wu, H.J., Kaizuka, Y., Vale, R.D., and Groves, J.T. (2010). Altered actin centripetal retrograde flow in physically restricted immunological synapses. PLoS One *5*, e11878.

OPEN
ACCESS
CellPress

STAR★METHODS

KEY RESOURCES TABLE

REAGENT or RESOURCE	SOURCE	IDENTIFIER
Antibodies		
Mouse anti-human CD3 (OKT3)	eBioscience	Cat#16-0037-81
Mouse anti-human CD3 (UCHT1)	eBioscience	Cat#16-0038-81
Chemicals, Peptides, and Recombinant Proteins		
HLA-A2 MHC	Aleksic et al., 2010	N/A
NY-ESO-1$_{156-157}$ 9V	Generon UK	N/A
NY-ESO-1$_{156-157}$ 4D	Generon UK	N/A
ICAM1, hIgG1-Fc.His Tag	Thermo Fisher	Cat#10346H03H50
DOPC	Avanti Polar Lipids	Cat#850375
Ni_2^+-NTA-DGS	Avanti Polar Lipids	Cat#790404
Cap biotin PE	Avanti Polar Lipids	Cat#870273
NaCl	Sigma-Aldrich	Cat#S9888
KCl	Sigma-Aldrich	Cat#P9541
Glucose	Sigma-Aldrich	Cat#G8270
$CaCl_2$	Sigma-Aldrich	Cat#449709
$MgCl_2$	Sigma-Aldrich	Cat#M8266
Casein	Sigma-Aldrich	Cat#C7078
$NiCl_2$	Sigma-Aldrich	Cat#339350
Streptavidin	Sigma-Aldrich	Cat#85878
Jasplakinolide	Sigma-Aldrich	Cat#J4580
Y27632	Sigma-Aldrich	Cat#Y0503
RPMI-1640	Sigma-Aldrich	Cat#R8758
FBS	Sigma-Aldrich	Cat#F9665
Human Serum Albumin (HSA)	Sigma-Aldrich	Cat#SRP6182
Penicillin-streptomycin	Sigma-Aldrich	Cat#P4333
L-glutamine	Lonza	Cat#17-605E
HEPES	Sigma-Aldrich	Cat#H0887
Sodium Pyruvate	Lonza	Cat#13-115E
Poly-L-Lysine	Sigma-Aldrich	Cat#P8920
EZ-Link Sulfo-NHS-LC-Biotin	Thermo Fisher	Cat#21335
Sulfo-SANPAH	Thermo Fisher	Cat#22589
Streptavidin-acrylamide	Thermo Fisher	Cat#S21379
Biotinylated Bovine Serum Albumin	Sigma-Aldrich	Cat#A8549
Bovine Serum Albumin	Sigma-Aldrich	Cat#A2153
CellMask Deep Red	Thermo Fisher	Cat#C10046
Cholestorol-PEG-KK114	Honigmann et al., 2014	N/A
40nm red (594/620) fluorescent beads	Invitrogen	Cat#F8793
SNAP-cell-505	NEB	Cat#S9103S
Glutaraldehyde	Sigma-Aldrich	Cat#340855
APTMS	Sigma-Aldrich	Cat#281778
APS	Sigma-Aldrich	Cat#A3678
TEMED	Sigma-Aldrich	Cat#T9281
N,N'-Methylenebisacrylamide solution (2%)	Sigma-Aldrich	Cat#M1533
Acrylamide solution (40%)	Sigma-Aldrich	Cat#A4058

(*Continued on next page*)

Continued		
REAGENT or RESOURCE	SOURCE	IDENTIFIER
Experimental Models: Cell Lines		
Jurkat 1G4-TCR T cell	Lee et al., 2017	N/A
Recombinant DNA		
Lifeact-mCitrine	Addgene	54733
Software and Algorithms		
ABAQUS 6.124-3	ABAQUS, Inc.	N/A
MATLAB 2018a	MathWorks, Inc.	N/A
Trackpy	N/A	https://soft-matter.github.io/trackpy/v0.3.2/)
PicoQuant SymPhoTime	PicoQuant	N/A

CONTACT FOR REAGENT AND RESOURCE SHARING

Further information and requests for reagents may be directed to and will be fulfilled by the Lead Contact, Marco Fritzsche (marco.fritzsche@rdm.ox.ac.uk).

EXPERIMENTAL MODEL AND SUBJECT DETAILS

A Jurkat T cell line stably expressing exclusively the 1G4 TCR was generating via viral transduction of a Jurkat T cell line containing no endogenous TCR, as described in Lee et al. (2017). 1G4 Jurkat T cells were cultured at 37°C in 5% CO_2 in RPMI-1640 (Sigma Aldrich, UK) containing 10% FBS (Sigma Aldrich), 1% Penicillin Streptomycin (Sigma Aldrich), 1% L-glutamine (GIBCO, UK), 10mM HEPES (Sigma Aldrich) and 1mM Sodium Pyruvate (GIBCO). Cells were split every two days at a volume ratio of 1:5.

METHOD DETAILS

pMHC Production

HLA-A2 molecules were synthesized as previously described (Aleksic et al., 2010). Two variants of the NY-ESO-$1_{157–165}$ peptide were presented on the HLA-A2 molecule; one with a mutation to valine at position 165 (referred to as 9V) and one with a mutation to aspartate at position 160 (referred to as 4D). Peptides were synthesized by Generon (UK).

Protein Biotinylation

Protein biotinylation was required to specifically functionalize glass, gel and bilayer surfaces. Biotylination of both antibodies and HLA-A2 molecules was achieved via amine reactive chemistry (EZ-Link Sulfo-NHS-LC-Biotin, ThermoFisher). All proteins to be biotinylated were first passed through a desalting column (7K Zeba Spin, ThermoFisher, UK). Sulfo-NHS-LC-Biotin was added to the 1 mg/ml protein solution at a molar ratio of 10:1 and incubated for 1 h at 37°C. Following incubation, the solution was passed through a further desalting column to remove any unbound biotin.

Bilayer Preparation

Bilayers were prepared using previously described protocols (Choudhuri et al., 2014; Grakoui et al., 1999). Briefly, for coupling of polyhistidine tagged ICAM-1 (Abcam, UK) and biotinylated anti-CD3 (OKT3), equal volumes of DOPC (Avanti, USA) liposomes (0.4 mM), and liposomes containing 12.5 mol% Ni_2^+-NTA-DGS (Avanti), 0.05 mol% cap biotin PE (Avanti) and 75 mol% DOPC (0.4 mM) were mixed and deposited onto multi-well flow-chambers (Ibidi, UK). Chambers were then washed with supplemented HEPES buffered saline (20mM HEPES, 140 mM NaCl, 5mM KCl, 6 mM glucose, 1mM $CaCl_2$, 2 mM $MgCl_2$, 1% human serum albumin (HSA), pH 7.2. Following blocking for 30 min with 5% casein supplemented with 100 μM $NiCl_2$, bilayers were incubated for 30 min with polyhistidine tagged ICAM-1 and streptavidin. Following extensive washing, bilayers were incubated with biotinylated OKT3.

PAA GEL PREPARATION

PAA gel preparation has been adapted from Tse et al. (2010) and described in detail in Colin-York et al. (2017). Specifically, 40% acrylamide (Sigma Aldrich) and 2% bis-acrylamide (Sigma Aldrich) solutions were combined at 1% and 0.1% respectively to make a 1 kPa gel and 4% and 0.3% to make a 3 kPa gel. Polymerization was initiated by adding ammonium persulfate (APS) (Sigma Aldrich) and tetramethylethylenediamine (TEMED) (Sigma Aldrich) at volume ratios of 1:100 and 1:250, respectively. Once initiated, the gel solution was quickly pipetted between two coverslips, forming a sandwich. Prior to forming the sandwich, the upper coverslip

was coated with a high density of fluorescent beads. To achieve a good coverage of fluorescent beads on the coverslip, the glass was first acid cleaned and then coated with poly-L-lysine 0.01% (Sigma Aldrich) for 10 min. After washing and drying, a solution containing a high concentration of 40nm red (594/620) fluorescent beads (Invitrogen) was placed onto the coverslip for 10 min, followed by washing and drying. The top coverslip was treated with 0.5% (3-aminopropyl)trimethoxysilane (APTMS) (Sigma Aldrich) followed by treatment with 0.5% glutaraldehyde (Sigma Aldrich) solution. This results in the silanisation of the glass surface which forms a covalent link with the polymerizing gel, assuring firm attachment of the underside of the gel to the coverslip. Once the sandwich was formed, the PAA gel was allowed to polymerize for 30 min at room temperature. Once complete, the upper coverslip was peeled off the gel, leaving a thin layer of gel on the activated surface. The PAA gel was then washed extensively in 100x vol/vol Phosphate-buffered saline (PBS) and stored at 4°C for no longer than 2 weeks. Gel stiffness was validated for each nominal stiffness using AFM indentation (Denisin and Pruitt, 2016) (Figure S1).

PAA Gel Functionalisation

PAA gel functionalisation was achieved by two different methods. One based on a biotin streptavidin interaction and the other on the bi-functional cross-linker Sulfo-SANPAH ((sulfosuccinimidyl 6-(4'-azido-2′-nitrophenylamino)hexanoate) (Thermo Fisher) in combination with 365 nm ultraviolet (UV) light. For the first method, gels were fabricated using the previously described method, with the addition of streptavidin-acrylamide (Thermo Fisher) to the gel solution at a final concentration of 750 μg mL^{-1}. As the gel polymerizes on addition of TEMED and APS the streptavidin-acrylamide is covalently incorporated into the meshwork of the gel leading to a distribution of biotin binding sites throughout the gel layer. After washing in PBS, the gel was placed on a drop of biotinylated protein solution, for example OKT3-biotin at a concentration of 100 μg mL^{-1}. Following 2 h incubation at room temperature, the gel was washed again in PBS and was then ready to use. Antigen concentration on the top surface of the gel was validated using the binding of the Streptavidin-488 to ensure the degree of coating was the same across all antigens (Figure S1). Alternatively, gels were functionalised using Sulfo-SANPAH. Gels were again fabricated as previously described. Once polymerized, the gels were coated with a 5 mg mL^{-1} solution of Sulfo-SANPAH and exposed to UV light at 365 nm for 10 min. Following this, the gel was extensively washed in PBS, ensuring all unbound cross-linker was removed. The gel was then placed on a drop of poly-L-lysine at a concentration of 1 mg mL^{-1} and incubated at 4°C for 12 h. Following incubation, the gel was washed in PBS. At this point the gel was incubated with a solution of OKT3 at a concentration of 100 μg mL^{-1}.

Coverslip Preparation

Glass coverslips functionalised with both anti-CD3 antibodies and NY-ESO pMHCs were required for actin velocity quantification. 18 mm, #1.5 coverslips (Scientific Laboratory Supplies, UK) were first sterilized by extensive washing in ethanol. After drying, coverslips were coated with 20 μL of a 1:4 mix of biotinylated Bovine Serum Albumin (BSA) (Sigma Aldrich) and standard BSA (Sigma Aldrich) at concentrations of 200 μg mL^{-1} and 800 μg mL^{-1} respectively, followed by incubation at room temperature for 2 h. After washing 3 times in PBS, coverslips were coated with 20 μL of a 10 μg mL^{-1} solution of streptavidin (Sigma Aldrich) followed by a further 2 h incubation. After washing, 20 μL of a 10 μg mL^{-1} solution containing either the biotinylated anti-CD3 antibodies, OKT3 or UCHT1, or the biotinylated NY-ESO pMHC, HLA-9V and HLA-4D was placed on each coverslip as required, followed by a further incubation 2 h and washing.

TFM data acquisition

TFM experiments were conducted using a Leica SP8 (Leica Microsystems GmbH, Mannheim, Germany) confocal microscope equipped with a Leica HC PL APO 63 × /1.20 NA water motCORR CS2, a white-light-laser (WLL, NKT Photonics) for flexible choice of excitation wavelengths, and environmental control maintaining a temperature of 37°C in an atmosphere of 5% CO_2. 1G4-TCR Jurkat T cell expressing actin-SNAP and labeled with SNAP-Cell-505 were allowed to interact with PAA gels functionalised with anti-CD3 antibodies or NY-ESO pMHCs as described previously. Bead displacements were recorded via time-lapse imaging at a laser power of 488 nm – 36 μW and 594 nm – 55 μW and a frame rate of 2 s^{-1} for 2-3 mins per cell.

TFM analysis

Bead displacement data was extracted from the confocal time-lapse imaging using a custom written MATLAB routine. For each tracked bead, in order to estimate forces transmitted to the PAA gel by cells, ABAQUS 6.124-3, a commercial software package for Finite Element (FE) analysis, was used to solve the boundary value problem. The PAA gel was simulated as a cube and displacements generated by cells were applied to the top surface of the cube.

Generally, three sets of equations are required to solve the boundary value problem:

1 The dynamic equilibrium equation which can be written as:

$$\frac{\partial \sigma_{ij}}{\partial x_j} + \rho f_i = \rho \ddot{u}_i \tag{1}$$

in which, σ_{ij} = stress tensor, x_j = right-handed Cartesian coordinates (i,j = 1,2,3), ρ = density of material, f_i = external body forces per unit volume and $\ddot{u}$ is the acceleration of the element. Since the force generation of the cells is dynamic in nature, a dynamic explicit formulation was used in our computation.

2 The constitutive relationship:

$$\sigma_{ij} = \frac{E\,\upsilon}{(1+\nu)(1-2\nu)}\varepsilon_{kk}\delta_{ij} + \frac{E}{1+\upsilon}\varepsilon_{ij} \tag{2}$$

where E = Young's modulus, υ = Poison's ratio, δ_{ij} = Kroneker delta and ε_{ij} = strain tensor. The gel was assumed to have an isotropic linear elastic behavior, with E = 3 kPa, and ν = 0.3.

3 The strain-deformation equation:

$$\varepsilon_{ij} = \frac{1}{2}\left(\frac{\partial u_i}{\partial x_j} + \frac{\partial u_j}{\partial x_i}\right) \tag{3}$$

where u = displacement vector. In the definition of strain tensor, second order derivatives are ignored, and hence strains are assumed to be small. However, it is possible to simulate large displacements in ABAQUS by activating geometry non-linearity. In such an approach, the node coordinates are updated after each time increment. Stability of the solution was ensured by choosing a sufficiently small time increment.

The boundary conditions on the displacement and stress are as follows: (1) $u_i = u_i^*$ at top face of gel, where u_i^* is the displacement obtained from microscopy, (2) $u_i = 0$ at the bottom surface of the gel and (3)$\sigma_{ij}n_j = 0$at side faces of the gel, where n_j is unit vector which is normal to the faces.

Equations (1), (2), (3) and the boundary conditions are combined using FE to obtain the following relationship:

$$M\ddot{u} + C\dot{u} + K\,u = F \tag{4}$$

in which, M, C, and K are mass, damping, and stiffness matrices, respectively. u and F are nodal displacement and external forces vectors, respectively. The over dot indicates the derivative with respect to time. This equation is solved by the software, and displacement vector field is calculated. Next, using Equations (3) and (2), strains and stresses are determined at Gauss integration points, respectively. Forces can then be calculated using Equation (4). For FE analysis, the hexahedron (brick) type element was used, with 8 corner nodes and eight inner Gauss integration points. Dimensions of the gel were sufficiently large such that no displacements were observed at the edges of the computational space.

Once the evolution of force was calculated for each beads position, the individual load-fail events were quantified in terms of the duration of the loading phase, distance traveled during the loading phase and the peak force generated before the failing event.

eTIRF-SIM microscopy

eTIRF-SIM microscopy of F-actin and TCR dynamics was achieved using a custom build system which has previously been described in detail in Li et al. (2015). 1G4-TCR Jurkat T cell expressing Life-Act Citrine were allowed to interact with and SLB functionalised with labeled anti-CD3 (OKT3) as described previously. Imaging was conducted at a temperature of 37°C in an atmosphere of 5% CO_2.

Optical flow analysis

Optical flow is technique commonly used in computer vision to establish the movement between video frames. Here, we apply optical flow to measure to velocity displacement of actin between frames of time-lapse imaging. To establish the flow velocity for each cell, we first find the optical flow between each frame using a pre-implemented MATLAB algorithm (Liu, 2009). This results in a displacement map linking the position of each pixel between frames. By dividing the displacement by the frame acquisition time, we achieve the actin flow velocity between each frame. This was done for all frames of the time-lapse and the results plotted in a histogram. To understand the complex flow of actin at the IS, the optical flow vector field was transformed from Cartesian to polar co-ordinates, with the origin at the center of the cell contact, allowing the radial and azimuthal velocity components to be independently analyzed.

TCR cluster tracking

TCR cluster tracking was performed using custom written code written in Python and based on the tracking library known as Trackpy (https://soft-matter.github.io/trackpy/v0.3.2/). Briefly, the algorithm first located circular features of a user defined size and intensity range in each frame of the time-lapse (*tp.batch*). Defining a minimum displacement between frames, and a minimum track length, the code linked individual localizations into tracks allowing the TCR velocity to be calculated (*tp.link_df* and *tp.filter_stubs*).

FRAP and bleach region tracking

FRAP experiments were conducted using a Leica SP8 confocal microscope equipped with a HC PL APO 100 × / 1.40 NA oil objective and environmental control maintaining a temperature of 37°C in and an atmosphere of 5% CO_2. For each FRAP acquisition, a 1 μm diameter circular bleach region was defined and following an initial bleach period at 100% laser intensity in both the actin (488 nm) and CellMask Deep Red (633 nm, Invitrogen) channel, the fluorescence recovery was monitored for a further 40 s at a frame rate of 2.2 s^{-1}. Further details for the optimization of FRAP experiments are presented in Fritzsche and Charras (2015).

Bleach region tracking was achieved using the same Leica SP8 confocal microscope. Again, a circular bleach region of 1 μm diameter was defined in the periphery of the lamellipodium. Following bleaching with 100% 488 nm laser light, the wider region surrounding the bleach region was imaged for 15 s at frame rate of 4.4 s^{-1}. Tracking of the bleach region was achieved via level set segmentation in MATLAB (Li et al., 2010) and the velocity of the bleach region was extracted by applying a linear fit to the displacement.

Fluorescence correlation spectroscopy

Confocal FCS measurements were acquired on a Leica SP8 confocal microscope equipped with a HC PL APO 100 × /1.40 NA oil objective. Prior to the FCS measurement, 1G4 TCR Jurkat T cells were loaded with either CellMask Deep Red, or Cholestorol-PEG-KK114 (Honigmann et al., 2014) at a concentration of 1 μg mL^{-1}. FCS recordings were directly controlled by the Leica LAS AF software, which communicates with the PicoQuant SymPhoTime (PicoQuant, Berlin, Germany) software and hardware as a pre-integrated FCS package in LAS AF. Fluorescent light was collected onto a single-photon-counting avalanche photo-diode (APD; Micro Photon Devices, PicoQuant) in the external port of the microscope. The APD signal was recorded with a time-correlated single-photon-counting (TCSPC) detection unit (Picoharp 300, PicoQuant). FCS data were fit with a two-dimensional (2D) diffusion model using the FoCuS-point fitting software (Waithe et al., 2016).

QUANTIFICATION AND STATISTICAL ANALYSIS

Quantification

Quantification procedures are given in the Method Details.

GENERAL STATISTICAL METHODS

For normally distributed data, the geometric mean and standard deviation was calculated, as denoted in the results and in the figure legends of Figures 1, 2, and 3. Statistical comparison of normally distributed data was carried out using an unpaired t test or one-way ANOVA and significance was denoted as $p < 0.05$ (*), $p < 0.01$ (**), $p < 0.001$ (***), and $p < 0.0001$ (****). Outliers outside the normally distributed data were not considered in the significance tests but are included in all plots. All statistical tests were calculated using GraphPad Prism 7.

Cell Reports, Volume *26*

Supplemental Information

Cytoskeletal Control of Antigen-Dependent

T Cell Activation

Huw Colin-York, Yousef Javanmardi, Mark Skamrahl, Sudha Kumari, Veronica T. Chang, Satya Khuon, Aaron Taylor, Teng-Leong Chew, Eric Betzig, Emad Moeendarbary, Vincenzo Cerundolo, Christian Eggeling, and Marco Fritzsche

Supplementary Figures

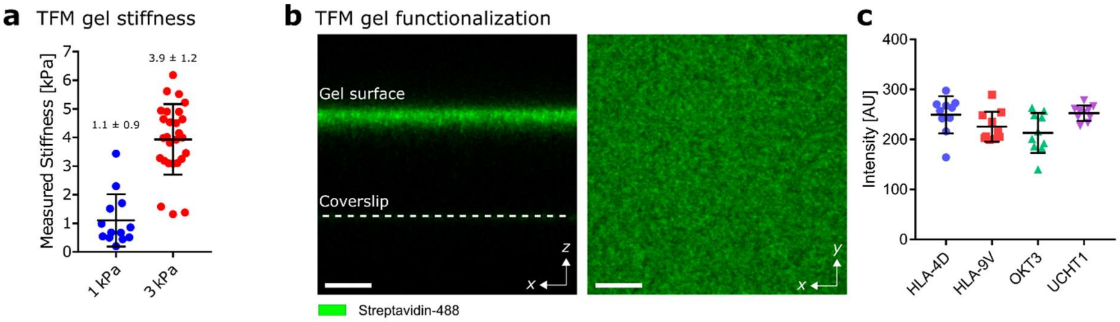

Figure S1 – **PAA Gel characterisation,** Related to Figure 1 - a) Stiffness of 1 kPa and 3 kPa PAA gel as measured by AFM indentation. b) Antigen coating on the PAA gel labelled by streptavidin 488. Scale bar is 10 µm. c) Quantification of antigen coating on the top surface of the gel. Scale bar is 5 µm.

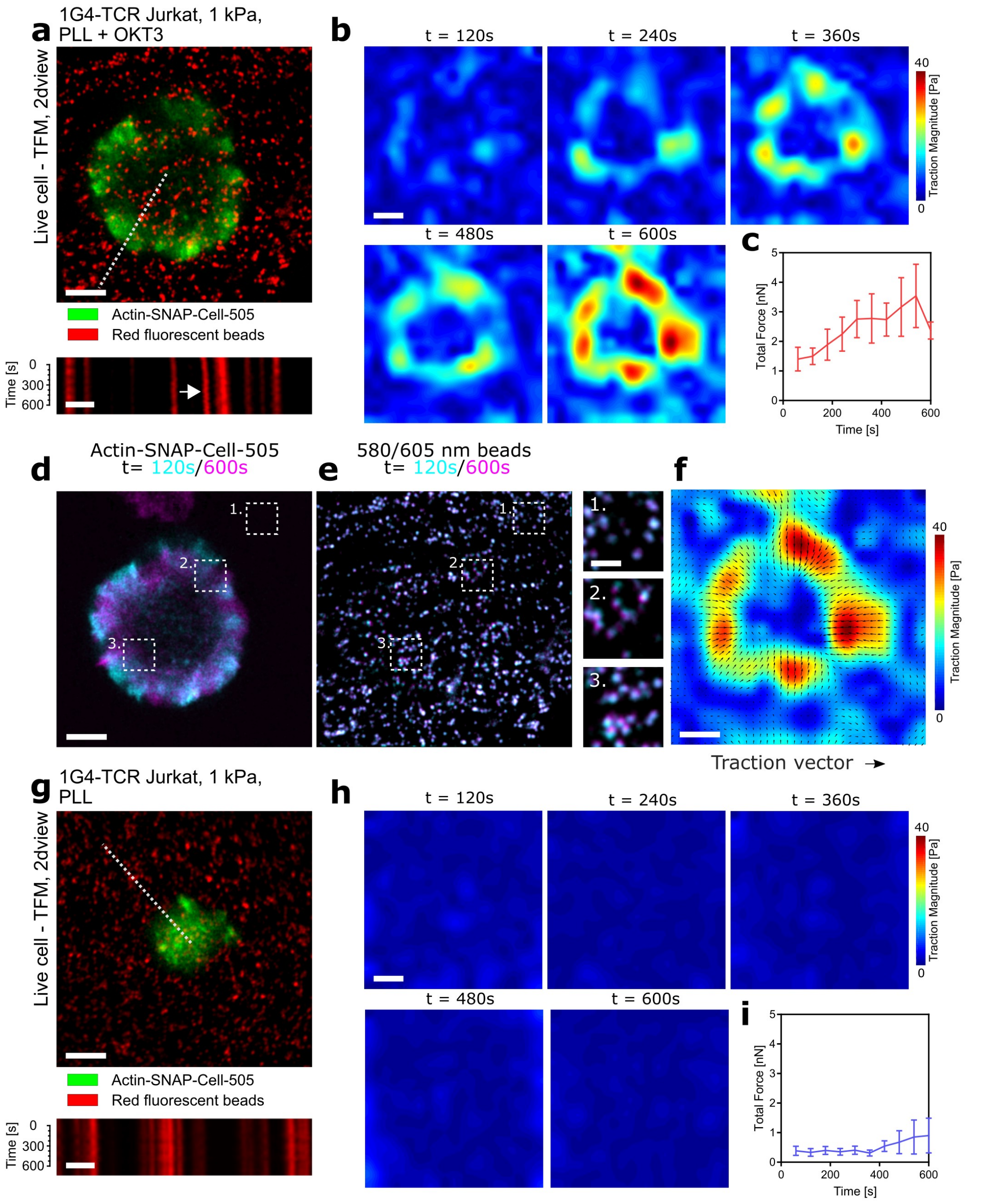

Figure S2 - **Force generation in the presence of OKT3 and PLL**, Related to Figure 1 - a) Upper - 1G4 TCR Jurkat T-cell interacting with 1 kPa PAA gel functionalised with PLL and OKT3. Scale bar is 5 µm. Lower – kymograph showing the gradual bead displacement during synapse maturation. Scale bar is 2 µm. b) Cumulative traction magnitude during the interaction, showing the gradual increase in force as the synapse matures in the presence of PLL and OKT3. Scale bar is 5 µm. c) Plot displays the integrated force over the whole contact area and again illustrates the gradual increase in the forces over the course of 600 seconds. d) Distribution of fluorescent actin during the early (cyan) and late (magenta) stage of synapse formation. Scale bar is 5 µm. e) Fluorescent bead distribution under the cell during the early (cyan) and late (magenta) stage of synapse formation. Inset dotted regions are shown on the right, and the lateral displacement of the beads is evident, showing the generation of force by the cell. Scale bar is 2 µm. f) Traction magnitude overlay with a traction vector field for the 1G4 TCR Jurkat T-cell interacting with 1 kPa PAA gel functionalised with PLL and OKT3. g) Upper - 1G4 TCR Jurkat T-

cell interacting with 1 kPa PAA gel functionalised with PLL only. Scale bar is 5 µm. Lower - kymograph showing minimal bead displacement during cell contact. Scale bar is 2 µm. h) Cumulative traction magnitude during the interaction, showing no force generation of the duration of the contact. Scale bar is 5 µm. i) Plot displays the integrated force over the whole contact area and again illustrates the lack of force generation over the course of 600 seconds.

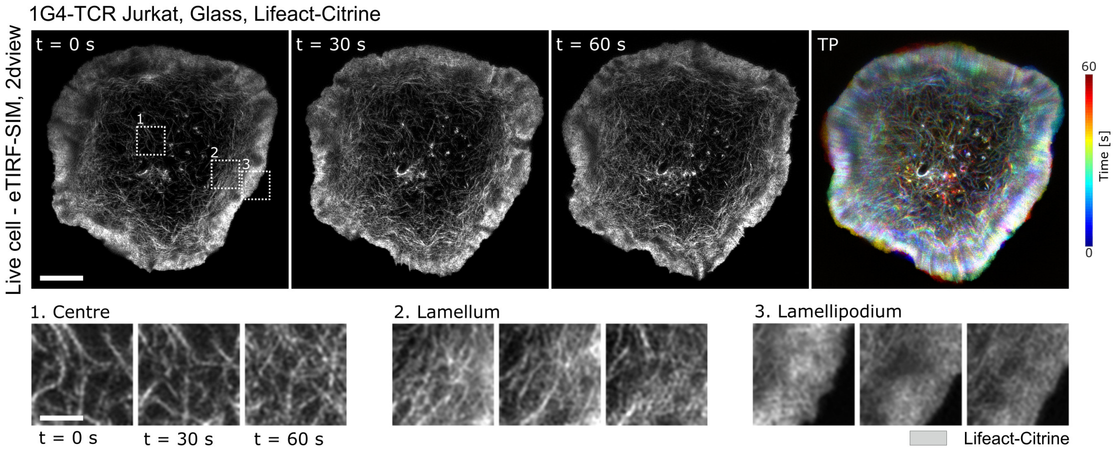

Figure S3 - **Actin dynamics imaged by eTIRF-SIM**, Related to Figure 2 – eTIRF-SIM timelapse imaging of 1G4-TCR Jurkat T-cells expressing Lifeact-**Citrine** interacting with antigen coated coverslips together with temporal projection (TP). Scale bar is 5 µm. Lower shows actin architecture in the central, lamellum and lamellipodial region of the cell. Scale bar is 1 µm.

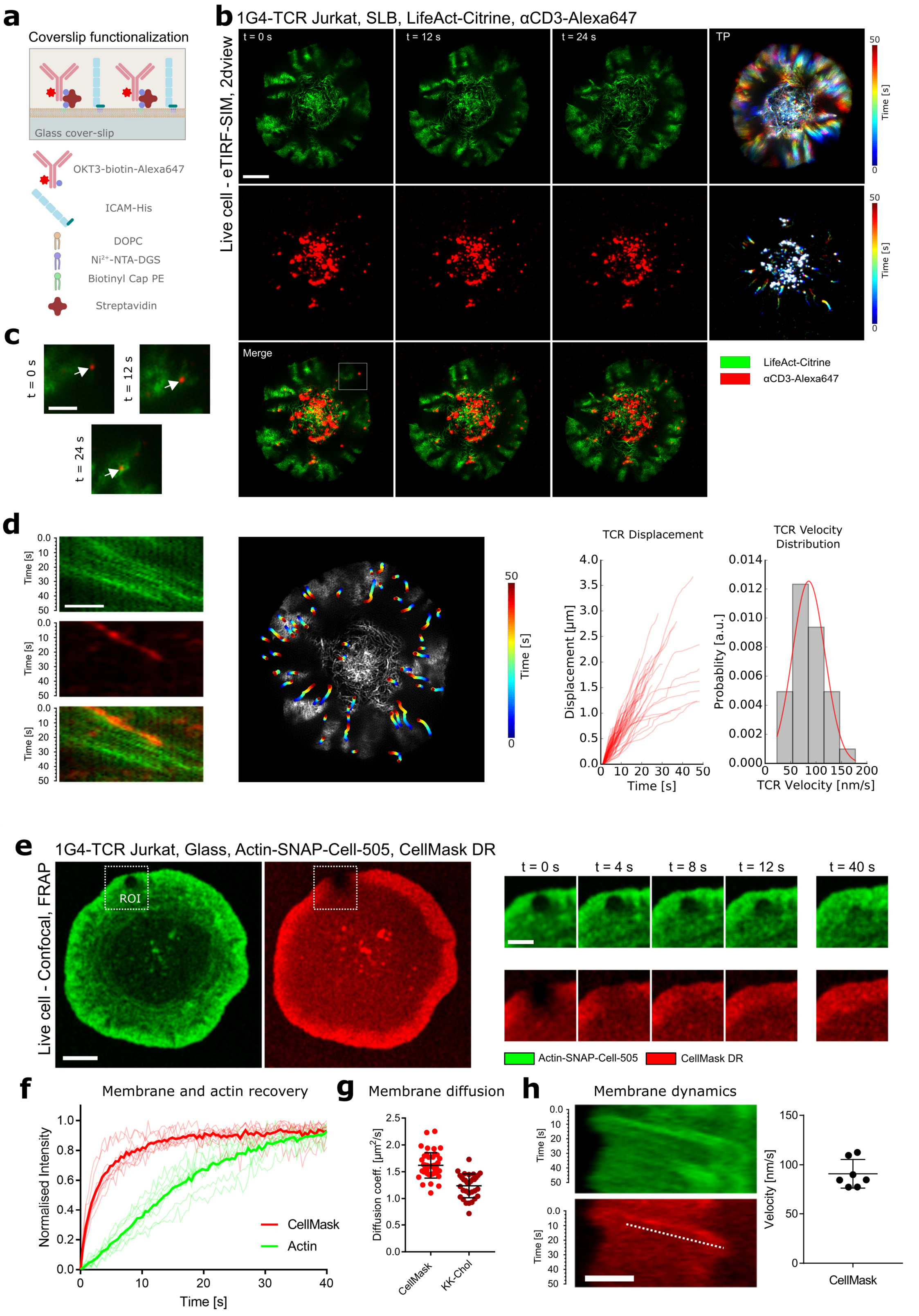

Figure S4 – **TCR cluster and membrane dynamics during T-cell activation,** Related to Figure 3 - a) Schematic outlining the functionalisation of SLB coated coverslips allowing stimulation via anti-CD3 antibodies. b) eTIRF-SIM fluorescent imaging time-lapse of 1G4-TCR Jurkat T-cell expressing Lifeact-citrine (green) interacting with

an SLB coated coverslips coated with anti-CD3-Alexa647 (red) and temporal projection (TP). Scale bar is 5 µm. c) Zoom in of dashed region in b) showing the movement of TCR micro-cluster as shown by the white arrow. Scale bar is 2 µm. d) Far left - Dual-colour kymograph showing the correlated motion of the actin cytoskeleton and TCR micro-clusters. Scale bar is 2 µm. Middle left – Single particle tracking of TCR micro-clusters during activation. Middle right – Quantification of TCR micro-cluster displacement. Far right - the distribution of TCR micro-cluster velocity with a mean of 85.5 ± 31.9 nm/s. e) Dual colour FRAP of actin (green) and plasma membrane (red) as labelled by SNAP-Cell-505 and CellMask Deep Red respectively during T-cell activation. Right – time-lapse imaging showing the recovery of the bleach region of actin (green) and plasma membrane (red). Scale bar is 2 µm. f) Quantification of the FRAP intensity recovery for actin and plasma membrane, highlighting the contrasting dynamics. g) Diffusion coefficient of the plasma membrane in the lamellipodium of an activating T-cell for the membrane dye CellMask Deep Red and labelled cholesterol (Chol-PEG-KK114) as measured by FCS. h) Kymograph analysis of actin and membrane dynamics showing correlative dynamics. Scale bar is 1 µm.
